# Antibacterial and antibiofilm activity of nanoparticles in *Klebsiella quasipneumoniae*

**DOI:** 10.1039/d5ra04828a

**Published:** 2025-11-10

**Authors:** Asha Devi, Rohit Kaundal, Neha Gupta, Deepak Kumar, Raman Preet Singh, Taranpreet Kaur

**Affiliations:** a School of Pharmaceutical Sciences, Shoolini University Solan HP 173 229 India ramanpreetsingh@hotmail.com rps@gpcpatiala.edu.in ashaa3456@gmail.com rohitkaundal68@gmail.com nehagupta5466@gmail.com guptadeepak002@gmail.com; b Department of Pharmacy, Government Polytechnic College Patiala PB 147 001 India; c Department of Biotechnology, Government Mohindra College Patiala PB 147 001 India tararnpreet.kaur@outlook.com +91-175-2321695

## Abstract

Bacterial biofilms are a complex, protective network comprising polysaccharides, proteins and nucleic acids which act as a physical barrier and are an important mechanism of antimicrobial resistance in human infections. Recent studies have highlighted potential of nanoparticles (NPs) as biofilm inhibitors hence, the present study evaluated biofilm inhibitory activity of clinically used metallic NPs (Ag, ZnO, TiO_2_) and carbon nanotubes (CNTs) in a biofilm-producing, clinical isolate *Klebsiella quasipneumoniae* ATCC 700603. A concentration-dependent reduction in biofilm production was observed with all NPs and was attributed to a reduction in bacterial viability. Additionally, ZnO also exhibited biofilm disrupting potential. Coarse-grained molecular dynamics revealed that NPs interacted with inner membrane, outer membrane, and peptidoglycan in decreasing order. Metallic NPs, particularly ZnO NPs, also interacted with a model biofilm. The results of the present study suggest possible therapeutic application of NPs in infection mitigation and control.

## Abstract 2

Bacterial biofilms are a complex, protective network comprising polysaccharides, proteins and nucleic acids which act as a physical barrier and are an important mechanism of antimicrobial resistance in human infections.

## Graphical abstract

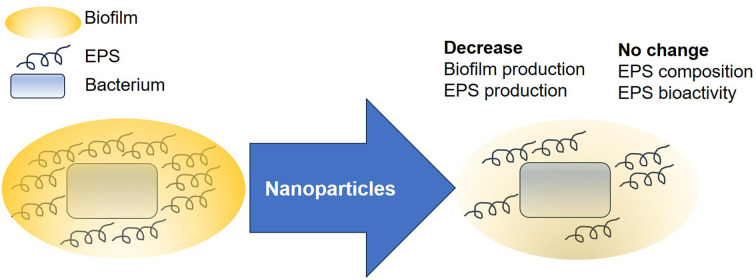

## Introduction

Antimicrobial resistance (AMR) has gained immense attention due to its omnipresence in pathogens. The magnitude of the problem can be well understood from the fact that World Health Organisation (WHO) initiated a Global Action Plan on AMR in 2015 and released a list of “priority pathogens” in 2017.^1^ The priority list of pathogens include pathogens for which development of new drugs, vaccines and diagnosis is required. The priority list is divided into 3 priorities (critical, high and medium) and carbapenem-resistant and extended spectrum beta-lactamase (ESBL)-producing members of family Enterobacteriaceae are in the critical category. This family of bacteria includes pathogens like *Escherichia coli* and *Klebsiella*.^2^ Traditionally, AMR has been attributed to point mutations and horizontal gene transfer however, in recent years, biofilms have attention due to their ability to confer resistance to a broad range of antimicrobial agents.^3^

Biofilms are produced as a defence mechanism against toxic compounds and desiccation while also providing ability to adhere and colonize biotic and abiotic surfaces. The biofilms are composed of polysaccharides, nucleic acids and proteins which are collectively called extracellular polymeric substances.^4^ The biofilm contributes to AMR by multiple mechanisms but the most important mechanism is by providing a physical barrier which restricts diffusion of the antimicrobial agent and reduced accessibility of the agent to bacteria. The biofilms also aid in horizontal gene transfer thus further hastening development of AMR. Hence, methods to inhibit biofilm production are an active area of research in drug discovery.^4,5^ The current interventions are mainly targeted towards polysaccharides in the biofilm thus highlighting their importance in biofilm structure and integrity. Small molecules which directly inhibit exopolysaccharide (EPS) synthesis or indirectly *via* inhibition of quorum sensing have been the major drug targets. Additionally, amylolytic enzymes capable of targeting EPS have also been investigated.^4–7^ Nevertheless, despite these advances, the repertoire of compounds targeting biofilm biosynthesis in general, and EPS in particular, have remained dismayingly low. This limitation in compounds targeting biofilms/EPS can to be attributed to a variety of reasons. First, the intracellular availability of the drugs is inhibited by biofilms as well as the cell wall and the two layers of membrane.^3,5,6^ Second, the use of enzymes for biofilm disruption pose significant challenges such as potential immunogenicity, narrow range of activity and impact on normal microbial biota.^8,9^ Third, different bacteria produce EPS with varying compositions using diverse sets of genes and targeting each of these biosynthetic pathways is not feasible.^10^ Further, bacteria can switch from producing one type of EPS to another thus adding another complexity in targeting biofilms.^11^ The need for biofilm inhibiting/disrupting compounds could be understood from the fact that 65% of bacterial infections and 80% of chronic infections are biofilm associated.^7,12^ EPS are an important component of biofilms with multifaceted functions. Interestingly, most bacteria produce only one or a few types of EPS which is regulated by expression of relevant genes and operons.^13,14^ The importance of EPS can be gauged from the fact that a vast proportion of anti-biofilm strategies are based on either inhibiting EPS biosynthesis or their hydrolysis.^7,15,16^ Studies with small molecule inhibitors have demonstrated that inhibition of biofilm production is often accompanied by changes in EPS polysaccharide composition.

Nanoparticles (NPs) have gained significant interest as anti-biofilm agents in recent years.^17^ A diverse range of carbon-based NPs, such as carbon nanotubes (CNTs) and graphene, and metallic NPs like silver, iron oxide, zinc oxide and other NPs as well as hybrids/composites containing more than one type of NP have been investigated for therapeutic and biofouling applications.^18–20^ When combined with drugs, NPs have been demonstrated to enhance antimicrobial activity and reverse AMR.^21–23^ Although NPs have been widely studied for their anti-biofilm properties, the reported studies have focussed on biofilm/EPS content only. Apparently, the effect of NPs on EPS composition has not been reported and attempts to understand the interactions between NPs and biofilm have only recently begun.^24^

NPs could also directly damage the microbial cells and result in bacterial killing. A wealth of literature reveals that carbonaceous, metallic and polymeric NPs could induce microbial killing by themselves as well as enhance activity of antimicrobial agents. Several of these studies have employed drug-resistant bacteria and demonstrated increase in activity of antimicrobial agents.^25^ Bacterial cell wall/cell membrane damage has been a prominent feature in several studies and studied using microscopy and biochemical assays. However, these studies provided limited information on nature of interactions given the complexity of cell wall and cell membrane.^25^ Molecular dynamics (MD) simulations, particularly coarse-grained (CG) simulations, have been extensively employed to study prokaryotic and eukaryotic lipid bilayers.^26,27^ However, interactions between bacterial membranes and NPs have not been extensively documented.^28,29^ Peptidoglycan (PG) is the major component of bacterial cell wall and plays a crucial role in maintaining structure and cellular homeostasis.^30^ Interestingly, despite its vital role, to the best of our knowledge, interactions between PG and NPs have not been investigated. We, therefore, investigated the interactions between bacterial membranes/PG and NPs using CG MD simulations. Apparently, a biofilm model has not been developed therefore, a CG model of biofilm was developed and interactions between NP–biofilm interactions were evaluated.

Towards this end, we employed *Klebsiella quasipneumoniae* ATCC 700603 (formerly *K. pneumoniae* K6) as a model organism. The choice of this organism was motivated by the fact that this organism is an ESBL-producing member of family Enterobacteriaceae and is thus in the critical category of WHO priority pathogens list. Secondly, this organism produces significant amounts of biofilm and has been widely employed as a model biofilm-producing pathogen.^31^ According to Nanotechnology Consumer Products Inventory,^32^ carbon nanotubes (CNTs), silver (Ag), titanium dioxide (TiO_2_) and zinc oxide (ZnO) NPs appear among top five NPs commonly encountered in consumer products^33^ and were hence included in this study.

## Experimental

### Bacteria and culture

*K. quasipneumoniae* ATCC 700603 (formerly known as *K. pneumoniae* K6) was generously provided by Venus Remedies Ltd, Panchkula, India. The cultures were maintained by subculturing on nutrient agar medium. Briefly, a colony of *K. quasipneumoniae* was picked with inoculation loop, suspended in sterile saline and plated by streaking on a fresh nutrient agar plate.

### Chemicals and culture media

Multi-walled CNTs (MWCNTs) were purchased from United Nanotech Innovations Pvt. Ltd, Bangalore, India. The MWCNTs had a length of 20 μm and external diameter 20–30 nm. The characterization of CNTs has been described in our earlier publications.^34,35^ Dulbecco's modified Eagle's medium (DMEM), fetal bovine serum (FBS), 3-(4,5-dimethylthiazol-2-yl)-2,5-diphenyltetrazolium bromide (MTT), Luria broth, nutrient agar, RPMI-1640 and crystal violet were purchased from HiMedia, Mumbai, India. All other chemicals were obtained either from Sigma, India or from local suppliers and were of the highest purity available.

### NP preparation, characterization and dispersion

**Fig. 1:**
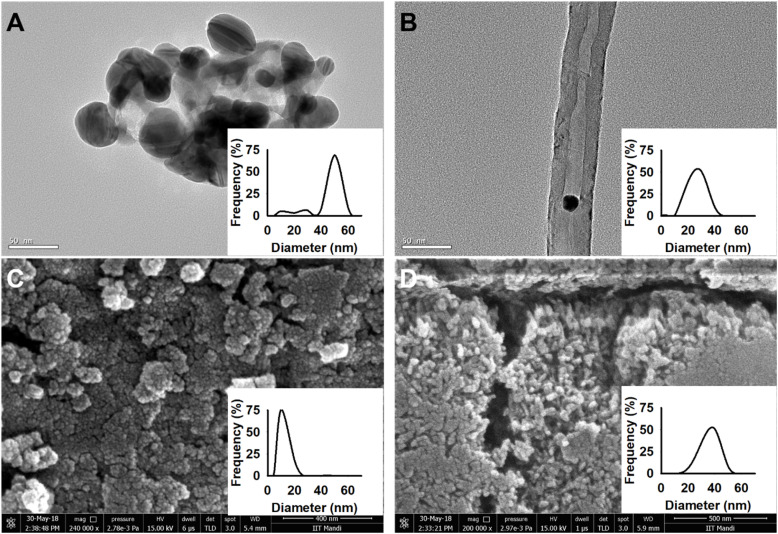
Electron microscopy of NP used in the study. (A) and (B) show HRTEM images of AgNP and MWCNT (B), respectively. (C) and (D) show SEM images of TiO_2_ and ZnO NPs, respectively. Scale bar is 50 nm in (A) and (B), 400 nm in (C) and 500 nm in (D). The inset shows diameter distribution determined from SEM/TEM images for 100–150 NPs except CNT where diameter of 75 CNTs was determined.

Citrate-capped AgNP were prepared by reduction of silver nitrate with trisodium citrate at ∼80 °C as described earlier.^36^ ZnO NPs were obtained by reaction of zinc nitrate with potassium hydroxide at room temperature followed by calcination at 500 °C.^37,38^ TiO_2_ NPs were synthesized at room temperature using titanium chloride and ethanol as precursor.^39^ Electron microscopy revealed that Ag and ZnO NPs were nearly spherical in shape with 30–50 nm diameter while TiO_2_ NPs were ∼10 nm in diameter. High resolution transmission electron microscopy (HRTEM) images of AgNP and MWCNT are shown in Fig. 1A and B, respectively. Scanning electron microscopy (SEM) images of TiO_2_ and ZnO NPs are shown in Fig. 1C and D, respectively. Dynamic light scattering analysis of freshly prepared aqueous suspensions (1 mg ml^−1^) was performed at room temperature.^40^ The hydrodynamic diameter (and polydispersity index) was found to be 51.6 nm (0.105), 21.5 (0.206) and 60.9 nm (0.177) for Ag, TiO_2_ and ZnO NPs, respectively.

MWCNTs (1 mg ml^−1^) were dispersed in 0.5% Tween-80 by probe sonication for 5 min as described earlier.^34,35,41^ The stock solution was serially diluted in broth to obtain desired working concentrations. This protocol was found to be effective in obtaining stable dispersions for atleast 72 h.^34,42,43^ AgNP aqueous suspensions were stable for several weeks as described in our previous report.^36^ ZnO and TiO_2_ NPs were dispersed in 1% Tween-80 as described for MWCNTs and suspensions were found to be stable for atleast 72 h.

### Preparation of NP dilutions

The NP stock (1 mg ml^−1^) was diluted 10-fold with Luria broth to obtain the highest working concentration of NPs (100 μg ml^−1^). This was further diluted by two-fold serial dilutions with Luria broth to obtain lower working concentrations. The working concentrations were added in triplicates in flat-bottom 96 well plates. The control wells either contained Luria broth or Tween-80 concentrations equivalent to those in CNT-containing wells.

### Antibacterial activity and biofilm assay

An overnight culture of *K. quasipneumoniae*, grown on nutrient agar at 37 °C, was selected and 4–5 colonies suspended in sterile saline. The optical density was adjusted between 0.6 to 0.8 at 600 nm with saline and 10 μl of inoculum was added to each well of the flat-bottom 96 well plate containing working concentrations of NPs and control (final volume 200 μl per well in Luria broth). After incubation for 72 h at 37 °C, the broth was aspirated and wells were washed thrice with water. In parallel experiments, 5 ml inoculum was added to 95 ml Luria broth in conical flasks with or without 100 μg ml^−1^ NPs and incubated for 72 h at 37 °C. Biofilm was extracted and biofilm components (protein, DNA and EPS) were quantified as described elsewhere with minor modifications.^44^ Briefly, the biofilm was dislodged by agitation of flask. The biofilm (50 ml) was then mixed with 200 ml of 36.5% formaldehyde and centrifuged (20 000*g*, 30 min, 4 °C). The pellet was resuspended in 10 ml of 1.5 M sodium chloride and again centrifuged (5 000*g*, 10 min, 4 °C). The supernatant was collected for estimation of biofilm component.^44^ Protein and EPS content were determined using Bradford assay and phenol-sulfuric acid method, respectively as described elsewhere.^44^ DNA was determined spectrophotometrically at 260 nm.^44^

MTT (0.5% w/v in Luria broth; 200 μl) was added in each well and incubated at 37 °C for 3 h, the dye was removed and formazon formed was dissolved in 20% w/v sodium dodecyl sulfate solution (100 μl per well) with mild sonication (30 s in a bath sonicator). The absorbance of dissolved formazon was measured at 595 nm and normalized to control assuming viability of control as 100%.^35^

Crystal violet dye (0.1% w/v in 20% v/v ethanol, 100 μl) was added in each well and incubated for 30 min at room temperature. The dye was removed, wells washed twice with distilled water and then air-dried at room temperature. The dye was solubilized by adding 95% v/v ethanol (100 μl) to each well and absorbance was measured spectrophotometrically at 595 nm using a microtiter plate reader.^45^ Biofilm production in control cells was considered 100% and biofilm produced in NP-treated cells was expressed as percent of control.

Biofilm disruption assay was performed as described elsewhere with minor modifications.^46^ Briefly, biofilm was allowed to grow for 72 h in 96 well plates as described for control wells in biofilm formation assay. After 72 h, the medium was aspirated and replaced with 200 μl fresh medium (control wells) or 200 μl fresh medium containing 100 μg ml^−1^ NPs. The plates were incubated for 6 h and then subjected to biofilm assay using crystal violet as described above. Biofilm content in control wells as considered 100%.

A parallel experiment was run in the same manner as above except that instead of MTT or crystal violet, the solvent of dye was added (Luria broth or 20% v/v ethanol) was added. The absorbance values obtained at 595 nm were <5% than that observed with crystal violet suggesting that NPs do not interfere in this colorimetric assay.^34,35^

### Cell wall/membrane integrity assays

*K. quasipneumoniae* inoculum was prepared as described above and 250 μl was inoculated in each well of flat-bottom 24 well plate containing 750 μl phosphate-buffered saline (PBS, pH 7.2). The plate was incubated with NPs (stock solution diluted in PBS) at a final concentration of 100 μg ml^−1^ and final volume was made to 1.5 ml per well with saline. After 3 hours, the contents of wells were centrifuged and absorbance at 260 and 280 nm was determined spectrophotometrically.^47^ In parallel control wells, only PBS was added in place of NP suspension.

*K. quasipneumoniae* were inoculated in black 96 well plates, incubated with 100 μg ml^−1^ NPs for 30 min and then propidium iodide (PI; final concentration 50 μM) was added in each well except that the medium used was 5 mM 4-(2-hydroxyethyl)piperazine-1-ethane-sulfonic acid (HEPES) containing 5 mM glucose and pH adjusted to 7.2. Fluorescence intensity was recorded at 580 nm excitation and 620 nm emission within 5 min of PI addition. Alternatively, *n*-phenyl-1-naphthylamine (NPN) was added in each well (final concentration 4 μM) and fluorescence intensity was recorded at 350 nm excitation and 420 nm emission within 5 min of NPN addition. The inoculum and NP working solutions were also prepared in the incubation medium.^48,49^

### Statistical analysis

The biofilm and cell viability assay data (absorbance values) was converted to percentages, with the average value in untreated, control wells representing 100%. The standard deviation of percentages was determined by Taylor's second moment expansion.^34^ The values were compared by One-Way analysis of variance (ANOVA) followed by *post hoc* Tukey's test using SigmaPlot program.

### Bilayer membrane models

The composition of bacterial inner membrane (IM) and outer membrane (OM) exhibit inter-species and intra-species (inter-strain) differences but significant inter-species similarities can still be identified among Gram-negative bacteria. Most of the experimental and computational studies on IM and OM have been performed using compositions identified in *Escherichia coli*. Since composition of *Klebsiella* sp. IM is not well characterized, the average composition of Gram-negative IM reported elsewhere^27^ was adopted in this study (SI Table S1). The average composition of Gram-negative IM shows remarkable similarities with *Klebsiella* sp. Whole cell lipid composition^50–52^ thus supporting relevance of the selected IM compositions. Similarly, the OM was also adopted from model Gram-negative OM^27^ where the outer leaflet is composed entirely of lipopolysaccharide (LPS) while the inner leaflet has a composition comparable to that of IM with minor differences. The Gram-negative IM and OM were originally described as all-atom (AA) models which were converted to coarse-grained models following MARTINI lipid definitions.^53,54^ The AA IM model contained significant proportion of cyclopropane fatty acids which are not described in the existing MARTINI lipid models and were thus modelled as saturated fatty acid chains using C1 beads as described elsewhere.^55,56^ The mapping scheme is shown in SI Fig. S1. The bonded parameters of cyclopropane lipids were optimized using AA model of QMPE (1-pentadecanoyl-2-*cis*-9,10-methylenehexadecanoic-acid-*sn*-glycero-3-phosphoethanolamine) as described in SI and optimised parameters listed in SI Table S2. More detailed information on parameterisation of cyclopropane lipids is included in SI Methods and results shown in SI Fig. S2 and S3.

The IM and OM were generated using CHARMM-GUI Martini Maker.^26^ Since CHARMM-GUI Martini Maker does not have parameters for cyclopropane lipids, the initial structures were generated using DPPE and DPPG and then modified to PMPE and PMPG, respectively. The IM was generated as a symmetric bilayer composed of 58/9/10/13/8/2 PMPE/POPG/PMPG/POPE/DOPE/CDL2 lipid bilayer and was enclosed in a box measuring 7.82 × 7.82 × 8.50 nm^3^ which also contained 67 Na^+^ and 21 Cl^−^ ions along with 1917 CG water molecules. The OM was composed of 35 LPS molecules (RAMP) in the outer leaflet and 75/20/5 POPE/PVPG/CDL2 in the inner leaflet. The OM lipid bilayer was enclosed in a box measuring 7.93 × 7.93 × 12.78 nm^3^ which also contained 61 Na^+^, 31 Cl^−^ and 175 Ca^2+^ ions along with 2644 CG water molecules.

The IM and OM were energy minimized using steepest descent algorithm (5000 steps) with position constraints applied on phosphate beads in the *z*-direction. The membrane was equilibrated by stepwise releasing position restraints (200, 100, 50, 20 and 10 kJ mol^−1^ nm^−2^ applied in the *z*-direction [membrane normal]) and simultaneously increasing timestep (2, 5, 10, 15 and 15 fs) in CG membranes. The timestep was kept 1 fs in all equilibration runs in AA QMPE membranes. The run times were 1 ns for the first three equilibration steps and 0.75 ns for the last two steps. This was followed by production run of 1 μs (for CG membranes, timestep 20 fs) or 200 ns (for AA membranes, timestep2 fs) without position restraints. Leap frog algorithm was used during equilibration and production runs while semiisotropic Berendsen^57^ and Parinello–Rahman barostat^58^ were used in equilibration and production runs, respectively. Membrane and water/ions were separately coupled to temperature bath and maintained at 310 K using velocity rescaling^59^ during equilibration and production runs. Verlet cut-off scheme^60^ was used for neighbour searching.

### PG model

PG is composed of glycan strands containing alternating *N*-acetylglucosamine and *N*-acetylmuramic acid residues with interstrand pentapeptide bridges. This general structure has been shown to be highly conserved in bacteria with minor modifications in peptapeptide composition and degree of interstrand linkages by pentapeptides. Experimental evidences suggest that a similar PG structure also exists in *K. pneumoniae*^61–63^ which is comparable to PG composition of *E. coli*.^64,65^ Hence, coarse-grained PG model of *E. coli* described elsewhere^30^ was used in the present study. The interactions between PG beads as well as between PG beads and NPs were scaled using the formula *ε*_scaled_ = 2 + *α*(*ε*_original_ − 2) where *α* = 0.7 except for water (P4) and ion (Qd) beads.^30,66^ The scaled force field (FF) parameters are available at https://www.github.com/ramanpsingh/Peptidoglycan-parameters/tree/main/KP. The PG network was energy minimised with position constraints (1000 kJ mol^−1^ nm^−2^) applied in the three directions. Equilibration runs of 1 ns each were carried out using semiisotropic Berendsen barostat (2 fs timestep) followed by Parinello–Rahman barostat with increasing timestep (5, 10 and 15 fs). This was followed by a production run with 15 fs timestep. The compressibility in xy direction was set to zero.^30^

### Biofilm simulations

The exact composition of *Klebsiella* sp. is variable and is composed of a large variety of proteins along with DNA and EPS. In a recent study on clinical isolates of *Klebsiella pneumoniae*, elongation factor Tu (TUF) was detected in biofilm samples. The structure of TUF protein from *K. quasipneumoniae* has not been characterised however, genome annotation of *K. quasipneumoniae* ATCC 700603 (GenBank ID CP014696.2)^67^ revealed the presence of TUF gene with inferred protein sequence (protein ID AMR13289.1) identical to *K. pneumoniae* (UniProt ID A6TEX7). The three-dimensional protein model of A6TEX7 was obtained from AlphaFold^68,69^ and converted to Martini 2.2 (ref. 70) model using CHARMM-GUI Martini Solution Maker module.^71^ The three-dimensional protein structure was maintained using elnedyn network.^72^ A 24-bp DNA structure was obtained from Martini website (https://www.cgmartini.nl) and converted to coarse-grained model using *martinize-dna.py* script.^73^ The structure of double-stranded DNA was maintained using soft elastic network. EPS isolated from *K. quasipneumoniae* ATCC 700603 has been shown to contain glucose, galactose, mannose and rhamnose linked by 1–2, 1–3 and 2–3 glycosidic bonds.^10^ Due to the coarse-grained nature of Martini FF, glucose and its epimers (mannose, galactose and rhamnose) are represented by the same Martini beads.^74^ The differences in glycosidic linkages will have an influence on the bonded parameters but not on non-bonded parameters (or bead types) hence, the qualitative nature of interactions will not be influenced. EPS was modelled as a polysaccharide containing 51 β(1–3)-linked glucose residues using an in-house python script (https://www.github.com/ramanpsingh/GlycanBuilder). The bonded parameters were based on those reported for laminaribiose and curdlan.^74^ Non-bonded parameters for proteins, DNA and EPS were those described in Martini 2.2 FF except that a scaling parameter of *α* = 0.7 was used between EPS beads^30,66,75^ as described above in PG model. The final biofilm was composed of 1 TUF molecule, 5 DNA molecules and 15 EPS molecules. The charge on the assembled biofilm was neutralised with Na^+^ ions, solvated with Martini water with approximately 10% anti-freeze water beads using *insane.py* script.^54^ The model was equilibrated in water using steps/parameters described for OM/IM/PG except that the simulation box was isotropic and coupled to stochastic cell rescaling barostat.^76^

### NP models

CG model of CNT was generated using python script described elsewhere (https://www.github.com/bio-phys/cnt-martini).^77^ The CNT model had 8 rings, each ring consisting of 8 beads, and bead was represented with CNP bead type.^78,79^ The CNT measured 1.2 nm in diameter and 2.8 nm in length.

AgNP were described by an implicit ligand model of citrate-coated NPs as described for gold NPs.^80^ Spherical NPs are solid spheres whereas the gold NP model employed a hollow sphere. The weight of the solid sphere was equally distributed in beads of the hollow sphere such that the total weight of hollow sphere remains equal to that of the solid sphere.^80^ Unit cell of silver was obtained from Crystallography Open Database^81,82^ (COD; https://www.crystallography.net/cod/1100136.html) and an atomic cluster of ∼2.5 nm diameter was constructed. The nanocluster contained 531 silver atoms while the CG model was composed of 126 beads. Hence, the mass of 531 silver atoms was distributed in 126 beads resulting in a bead weight of 454.59 in AgNP. Citrate binding to AgNP has not been widely studied and estimates obtained from thermogravimetric analysis reveal surface coverage of 12 to 46 citrate molecules per nm^2^ AgNP depending on concentration of citrate used for AgNP synthesis.^83^ Citrate molecules were found to occupy an area of 0.35–0.40 nm^2^ on silver sol (containing AgNP) resulting in surface coverage of ∼3 citrate molecules per nm^2^.^84,85^ Considering this variation in number of citrate molecules bound on AgNP surface, AA simulations were performed to determine number of citrate ions sorbed, and consequent surface charge, on AgNP surface. The detailed methods are available in SI methods and results shown in SI Fig. S4–S6 and Table S3. Based on surface coverage of 1.5 citrate molecules per nm^2^ of NP (obtained from AA simulations) and deprotonation state of ∼2.6,^80^ the net charge on AgNP was adjusted to −80*e*.

**Fig. 2:**
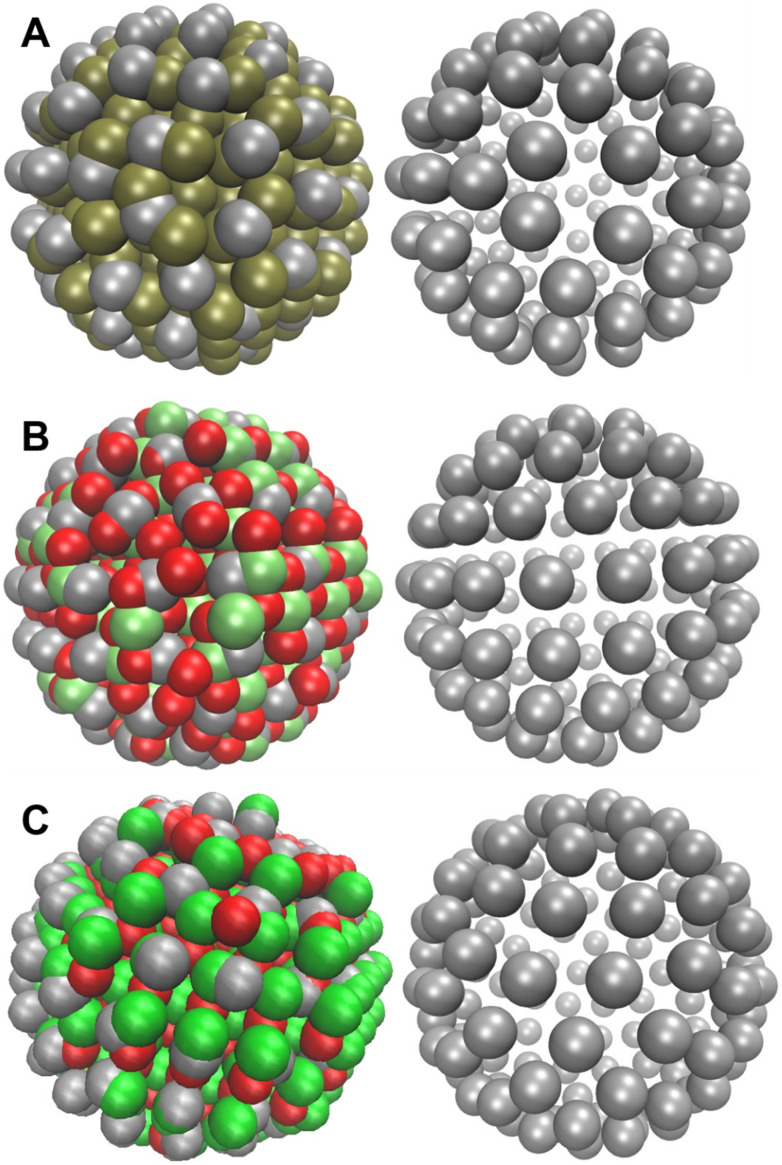
Models of Ag (A), TiO_2_ (B) and ZnO NPs (C). The left panel shows an overlay of all-atom and CG model while the right panel shows only the CG model. The red and green beads in the left panel represent metal and oxygen atoms, respectively. The silver beads in the left panel show the overlaid CG model shown separately in the right panel. All NPs have a diameter of ∼2.5 nm.

Unit cells of ZnO (COD ID: 1011259) and TiO_2_ (COD ID: 1526931) were obtained from Crystallography Open Database and nanoclusters were generated as described for AgNP. ZnO nanocluster had a total of 673 atoms and had the formula Zn_350_O_323_. TiO_2_ nanocluster had a total of 784 atoms with formula Ti_257_O_527_. Redistributing the mass of nanocluster to 126 beads results in bead weights of 211.99 and 164.51 for ZnO and TiO_2_ beads. In an all-atom MD study, a 4 nm TiO_2_ NP was modelled^86^ which had a total charge of −50*e*. In a later Monte Carlo simulation study, the authors developed a coarse-grained model of 4 nm diameter anatase TiO_2_ NPs assigning 5.2 beads per nm^2^ and charge of −0.19*e* per surface bead.^87^ This results in a charge of ∼ −50*e*. Assuming that surface charge density is not significantly altered by change in NP size,^87^ we assigned a total charge of −20*e* to ∼2.5 nm diameter NPs used in the present study. On a similar note, we constructed a ∼4 nm diameter ZnO NP and assigned partial charges according to Michaelis *et al.*^88^ and obtained a total charge of ∼+50*e*. Therefore, a surface charge of +20*e* was distributed on ∼2.5 nm diameter NP. CG MARTINI FF typically assigns integer charges to beads unlike all-atom FF which assign fractional charges. Therefore, in CG models of TiO_2_ and ZnO, a charge of −1*e* and +1*e*, respectively, were randomly assigned to 20 beads to obtain overall surface charge of −20*e*/+20*e* on hollow spheres as employed for AgNP. The CG models of the three NPs, each having a diameter of ∼2.5 nm, along with corresponding atomistic models of the same size are shown in Fig. 2.

CG models of metal oxide (TiO_2_ and ZnO) NPs are difficult to parameterize because, unlike small molecules and ions which exist as individual entities, metal oxide NPs exist as extensive 3-dimensional networks. Hence, routine parameterization schemes like free energy of vaporization and partitioning can not be used. In such cases, interfacial phenomenon such as interfacial tension^53^ and contact angle^89^ are routinely employed for parameterization. Since Martini beads are parameterized based on their hydrophilicity/hydrophobicity, we believe that AgNP and metal oxide NPs used in the present study could be described by Martini bead types. Based on CG simulations aimed at determining water contact angle, Ag, TiO_2_ and ZnO NPs were assigned C2, C2 and C1 bead types, respectively. The details of CG simulations and rationale for bead assignment are described in SI and results of contact angle simulations are shown in SI Fig. S7.

### MD simulations

NPs were introduced in a simulation box containing equilibrated OM, IM, PG or biofilm and solvated with approximately 90% water beads (W) and 10% anti-freeze water beads (WF). The charge was neutralised and osmolarity adjusted to 0.1536 M using Na^+^/Cl^−^ ions. The solvation and ion introduction steps were performed using *insane.py* script.^54^ The systems were then energy minimised and equilibrated followed by production run of 1 μs using steps/parameters described above for OM/IM/PG/biofilm depending on the biomolecule(s) in the system. The final compositions are summarised in Table 1. MD simulations were performed using Gromacs MD engine^90^ and Martini 2.2 FF.^53,91^ MD input files and trajectories were visualised using Visual Molecular Dynamics (VMD) program.^92^

**Table 1:** MD simulation systems

Target	NP^e^	Solvent and ions	Box size (nm^3^)^f^
OM^a^	AgNP	W: 2821, WF: 313, Na^+^: 110, Cl^−^: 0, Ca^2+^: 175	7.7 × 7.7 × 15.1
	CNT	W: 2879, WF: 319, Na^+^: 51, Cl^−^: 21, Ca^2+^: 175′	7.7 × 7.7 × 15.1
	TiO_2_	W: 2829, WF: 314, Na^+^: 60, Cl^−^: 10, Ca^2+^: 175	7.7 × 7.7 × 15.1
	ZnO	W: 2829, WF: 314, Na^+^: 40, Cl^−^: 30, Ca^2+^: 175	7.7 × 7.7 × 15.1
IM^b^	AgNP	W: 2853, WF: 317, Na^+^: 126, Cl^−^: 0	7.8 × 7.8 × 12.2
	CNT	W: 2946, WF: 327, Na^+^: 60, Cl^−^: 14	7.8 × 7.8 × 12.2
	TiO_2_	W: 2945, WF: 327, Na^+^: 70, Cl^−^: 4	7.8 × 7.8 × 12.2
	ZnO	W: 2945, WF: 327, Na^+^: 50, Cl^−^: 24	7.8 × 7.8 × 12.2
PG^c^	AgNP	W: 13 083, WF: 1453, Na^+^: 295, Cl^−^: 33	13.6 × 12.2 × 12.5
	CNT	W: 13 153, WF: 1461, Na^+^: 256, Cl^−^: 74	13.6 × 12.2 × 12.5
	TiO_2_	W: 13 109, WF: 1456, Na^+^: 265, Cl^−^: 63	13.6 × 12.2 × 12.5
	ZnO	W: 13 109, WF: 1456, Na^+^: 245, Cl^−^: 83	13.6 × 12.2 × 12.5
Biofilm^d^	AgNP	W: 49 214, WF: 5468, Na^+^: 780, Cl^−^: 454	21.3 × 14.6 × 23.6
	CNT	W: 49 296, WF: 5477, Na^+^: 741, Cl^−^: 495	21.3 × 14.6 × 23.6
	TiO_2_	W: 49 210, WF: 5467, Na^+^: 750, Cl^−^: 484	21.3 × 14.6 × 23.6
	ZnO	W: 49 210, WF: 5467, Na^+^: 730, Cl^−^: 504	21.3 × 14.6 × 23.6

a Outer membrane (OM) was asymmetric and contained only LPS in the outer leaflet (35 RAMP) and phospholipids in the inner leaflet (75 POPE, 20 PVPG, 5 CDL2).

b Inner membrane (IM) was symmetric and composed of 100 phospholipid molecules (58 PMPE, 9 POPG, 10 PMPG, 13 POPE, 8 DOPE, 2 CDL2) each in inner and outer leaflet. The lipid definitions described elsewhere^26,54^ and in SI (Table S1) were adopted for OM and IM and are: CDL2 = cardiolipin with -2*e* net charge; POPE = palmitoyl-oleoyl phosphatidylethanolamine; DOPE = dioleoyl phosphatidylethanolamine; PMPG = 1-palmitoyl-2-*cis*-9,10-methylene-hexadecanoicacid-phosphatidylglycerol; PMPE = 1-palmitoyl-2-*cis*-9,10-methylene-hexadecanoicacid-phosphatidylethanolamine; POPG = palmitoyl-oleoyl phosphatidylglycerol; PVPG = palmitoyl-vaccenyl phosphatidylglycerol; RAMP = Ra LPS.

c A single peptidoglycan (PG) network described elsewhere^30^ was used.

d Biofilm was composed of 1 protein (TUF1), 5 DNA (24 bp) and 15 EPS (51-mer) molecules.

e A single NP was introduced in the simulation box.

f The box dimensions represent size at the solvation step. The box boundary was atleast 1.5 nm away from any atom in target or NP.

## Results and discussion

### Antibacterial activity and biofilm assay

**Fig. 3:**
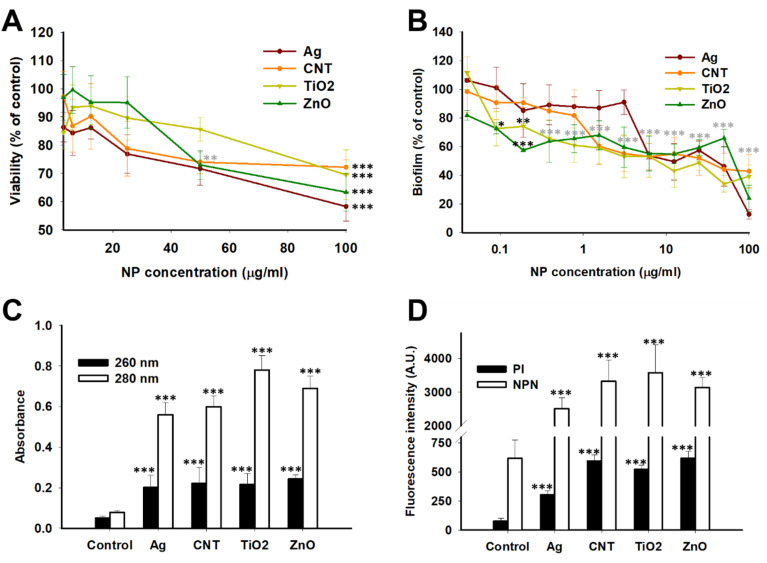
Effect of NPs on *K. quasipneumoniae* viability (A), biofilm production (B), cell wall/membrane integrity (C) and inner and outer membranes (D). Viability and biofilm production were determined following 72 h incubation with NPs by MTT and crystal violet assay, respectively. Cell wall/membrane integrity was determined following 3 h incubation with NPs by monitoring release of substances absorbing at 260 nm ad 280 nm. Inner and outer membrane damage were determined following 30 min incubation with NPs by PI and NPN uptake, respectively. NP concentration in (B) is expressed on a logarithmic scale. The fluorescence intensity in (D) is expressed in arbitrary units (A.U).

Bacterial cell viability and biofilm production in *K. quasipneumoniae* ATCC 700603 was determined following 72 h incubation with NPs. As shown in Fig. 3A, all NPs except TiO_2_ showed a significant (*p* < 0.01) reduction in cell viability at 50 μg ml^−1^. The cell viability was significantly (*p* < 0.001) reduced at the highest NP concentration (100 μg ml^−1^) and revealed that AgNPs were most effective followed by ZnO NPs while CNTs and ZnO NPs were equally effective. Further, all NPs showed a significant reduction in biofilm production compared to control (Fig. 3B). All NPs exhibited 50% reduction in biofilm production at 3.12–6.25 μg ml^−1^ however comparison at the highest NP concentration (100 μg ml^−1^) revealed that AgNPs were most effective followed by ZnO NPs while CNTs and ZnO NPs were equally effective. The order of reduction in biofilm production mirrored that observed in cell viability assays and suggest that a reduction in cell viability could be the determining factor in reducing biofilm production. The results agree with earlier studies on similar NPs^93–95^ thus confirming anti-microbial and biofilm-inhibiting properties of these NPs. Therefore, the mechanism of cell death and reduction in biofilm content were further investigated.

Data is mean ± SD (*n* = 4–6/concentration) and expressed as percent biofilm produced compared to control.

*p < 0.05, **p < 0.01, ***p < 0.001 compared to control (0 μg ml^−1^ NP) determined by One-way ANOVA and *post hoc* Tukey's test. In cases where data points are closely spaced or overlapping, a single set of symbols is placed above all such data points and the symbols are colored grey. The symbols for a single data point are in black.

### Effect on membranes and cell wall

Bacterial cell viability results (Fig. 3) show that NPs can reduce bacterial cell counts resulting in reduced biofilm production. Since the bacterial cell wall and membranes are key structures responsible for maintaining homeostasis and prevent the cell from exogenous toxic compounds, we hypothesised that NPs can interact with these structures and influence cell viability. Gram-negative bacteria, like *Klebsiella* sp., have a plasma membrane, commonly called inner membrane (IM), which is composed of phospholipids. The cell exterior is composed of another membrane, commonly called outer membrane (OM), whose outer leaflet is composed of LPS while inner leaflet is composed of phospholipids. The cell wall, composed of peptidoglycan (PG), is sandwiched between IM and OM.^26,27,96^ Experimentally, NPs have been demonstrated to induce anti-bacterial activity by cell wall and membrane damage as well as by stimulation of free radical production.^25^ We, therefore, studied leakage of intracellular components as a surrogate for cell wall/membrane damage. All NPs showed increased release of substances absorbing at 260 nm (nucleic acids) and 280 nm (proteins) suggesting that cell wall/membrane integrity was compromised. The absorbance values at 260 and 280 nm were 0.20–0.25 and 0.56–0.78 in NP-treated cells (100 μg ml^−1^, 3 h) while absorbance values were <0.1 at both wavelengths in control cells (Fig. 3C). This was accompanied by increased fluorescence of PI and NPN (Fig. 3D) suggesting damage to IM and OM, respectively.^48^

The experimental results were further complemented with unbiased MD simulation using CG Martini 2.2 FF^53^ to elucidate the nature of interactions between NPs and cell wall/IM/OM. The distance between membrane/PG and NP and number of contacts between membrane/PG and NPs were used to assess the degree of interactions.

**Fig. 4:**
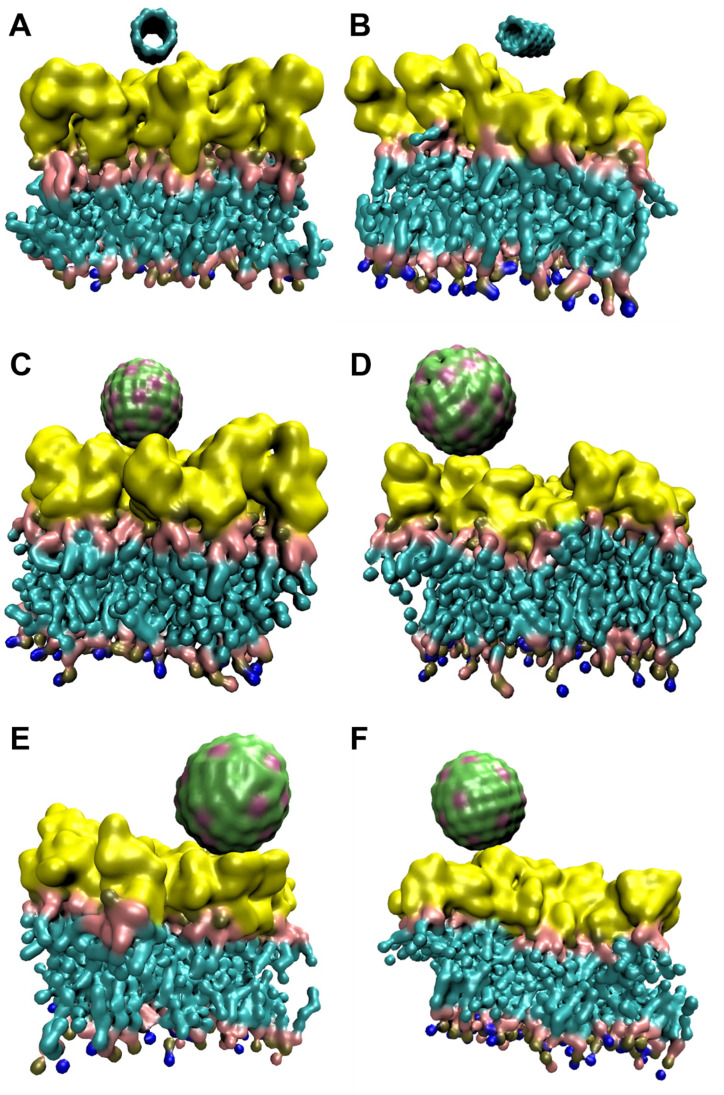
Trajectory snapshots showing interaction between OM and NPs. (A) and (B) show initial (0 μs) and final (1 μs) trajectory snapshot of interaction between OM and CNT, respectively. (C) and (D) show initial (0 μs) and final (1 μs) trajectory snapshot of interaction between OM and AgNP, respectively. (E) and (F) show final (1 μs) trajectory snapshot of interaction between OM and TiO_2_ NP and ZnO NP, respectively. The initial position of TiO_2_ and ZnO NPs were same as that of AgNP and are not shown. The yellow color corresponds to the outer leaflet comprising of glycan part of LPS while cyan colored region represent the lipid tails of LPS and inner leaflet. Water and ions are not shown for clarity. The lateral dimensions of box were approximately 7.7 nm × 7.7 nm.

**Fig. 5:**
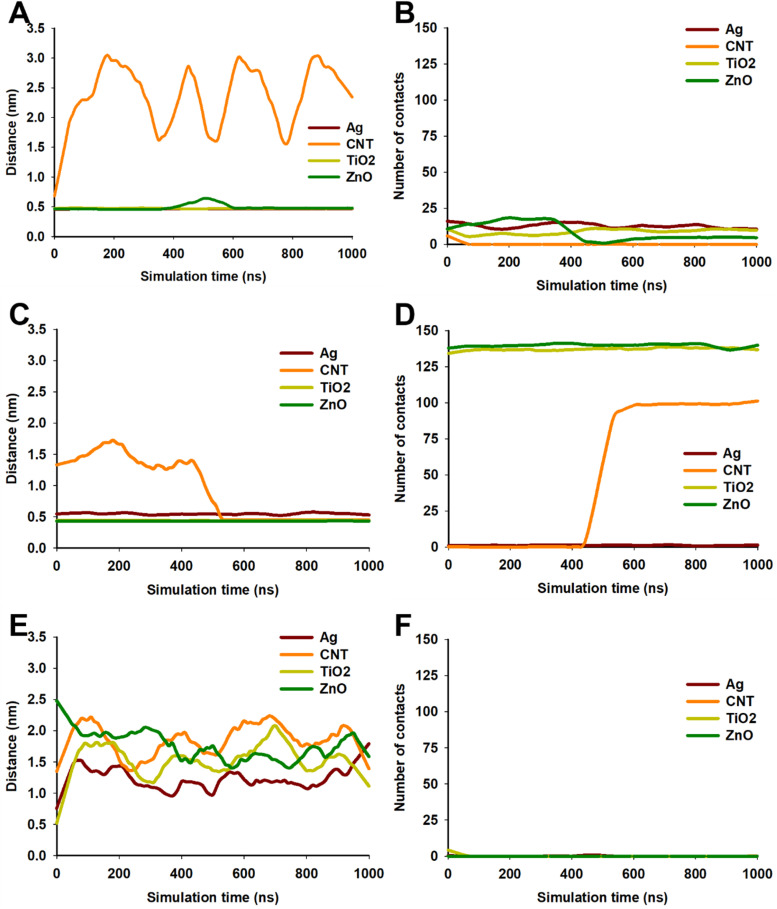
Interaction of NPs with OM (A and B), IM (C and D) and PG (E and F). A, C and E show distance of NP from OM, IM and PG, respectively, during 1 μs of simulation. B, D and F show number of contacts between NP and OM, IM and PG, respectively, during 1 μs of simulation.

As observed in Fig. 4, none of the NPs was able to penetrate the external LPS leaflet of OM during 1 μs simulation. However, striking differences were observed when distance and number of contacts between OM and NPs was observed over 1 μs simulation (Fig. 5). CNT showed least interactions with OM as the distance was >1 nm during most of the simulation (Fig. 5A). This was also supported by the low number of contacts between CNT and OM (Fig. 5B). CNT appeared to remain in proximity of OM up to the initial ∼20 ns and then moved away from OM suggesting a repulsive interaction. On the other hand, AgNP and TiO_2_ remained in constant contact with OM as evident from separation distance of ∼0.5 nm. AgNP appeared to better interact with OM (10–15 contacts) compared to TiO_2_ NP (5–10 contacts) throughout the simulation. ZnO NP exhibited relatively lower interactions as the separation distance from OM increased after 400 ns with a decrease in number of interactions. The outer leaflet of OM is composed of LPS molecules which are oriented in such a way that the lipid core of LPS forms the asymmetric bilayer while the saccharides orient towards the cell exterior thus contributing to hydrophilicity of the outer leaflet of OM.^26,96^ The presence of phosphate and carboxylic groups in LPS further contributes to its hydrophilic character. Since CNTs are hydrophobic in nature, their lack of interaction with OM is expected. On the other hand, the metallic NPs are all charged which impart some degree of hydrophilicity to the NP resulting in relatively higher interactions with LPS/OM.

**Fig. 6:**
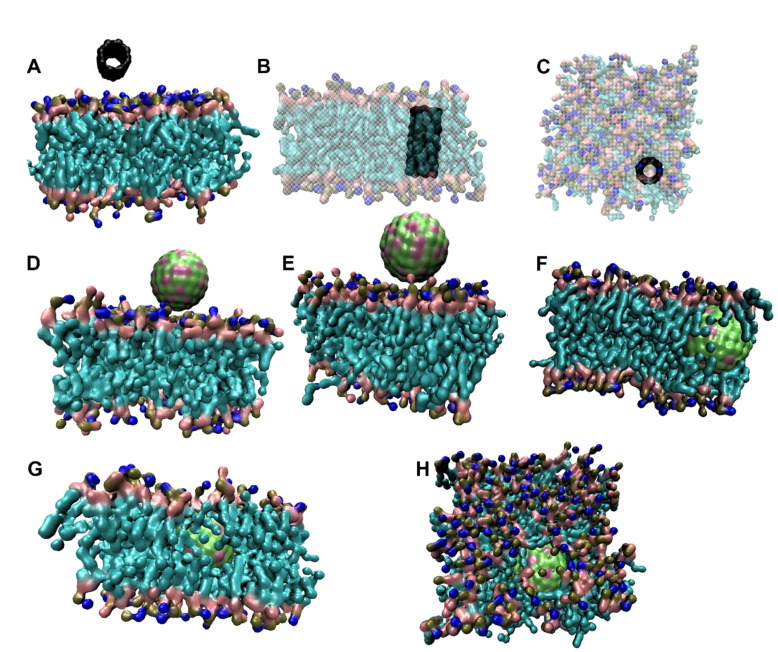
Trajectory snapshots showing interaction between IM and NPs. (A) and (B) show initial (0 μs) and final (1 μs) trajectory snapshot of interaction between IM and CNT, respectively. (C) Shows top view of (B). IM is shown transparent in (B) and (C) for better visibility of CNT inserted in IM. (D) and (E) show initial (0 μs) and final (1 μs) trajectory snapshot of interaction between IM and AgNP, respectively. (F) and (G) show final (1 μs) trajectory snapshot of interaction between IM and TiO_2_ NP and ZnO NP, respectively. (H) Shows top view of (G). The initial position of TiO_2_ and ZnO NPs were same as that of AgNP and are not shown. Water and ions are not shown for clarity. The lateral dimensions of box were approximately 7.8 nm × 7.8 nm.

**Fig. 7:**
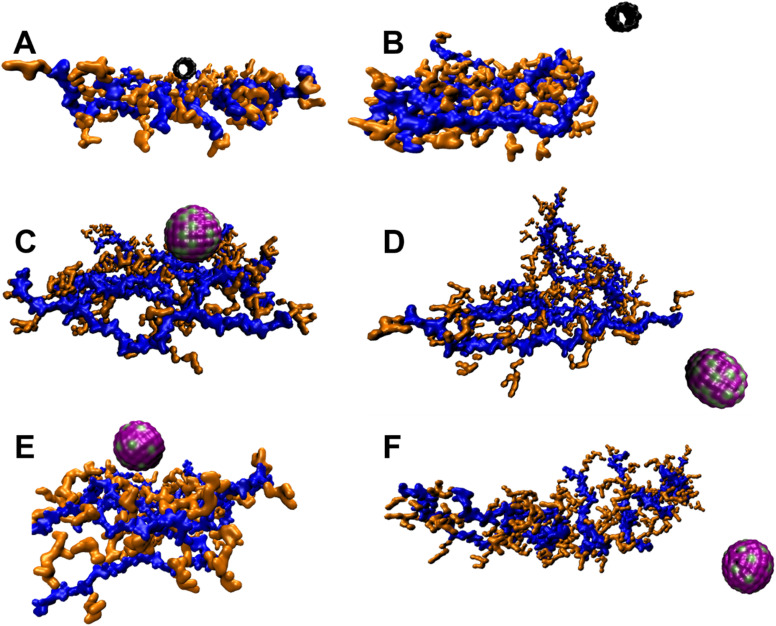
Trajectory snapshots showing interaction between PG and NPs. (A) and (B) show initial (0 μs) and final (1 μs) trajectory snapshot of interaction between PG and CNT, respectively. (C) and (D) show initial (0 μs) and final (1 μs) trajectory snapshot of interaction between PG and AgNP, respectively. (E) and (F) show final (1 μs) trajectory snapshot of interaction between PG and TiO_2_ NP and ZnO NP, respectively. The initial position of TiO_2_ and ZnO NPs were same as that of AgNP and are not shown. The blue color corresponds to glycan part while orange colored region represent the peptide linkers in PG. Water and ions are not shown for clarity. The lateral dimensions of box were approximately 13.6 nm × 12.2 nm.

In contrast to OM, CNT interacted efficiently with IM. As can be observed in Fig. 6, CNT inserted into IM during the course of simulation and remained in the IM until 1 μs of simulation. Fig. 5C shows that CNT made multiple contacts with IM and finally inserted in the IM after ∼500 ns as evident from a sharp (and stable) decrease in CNT-IM distance (Fig. 5C) with a simultaneous increase in number of contacts (Fig. 5D). In contrast to CNT, AgNP remained in close contact with IM but was not able to penetrate into the bilayer membrane (Fig. 5C, D, 6D and E). On the other hand, TiO_2_ and ZnO NP exhibited efficient interactions with IM and penetrated into the membrane (Fig. 6F–H). Both metal oxide NPs remained in close proximity of IM (<0.5 nm) throughout the simulation (Fig. 5C) and showed rapid insertion into the membrane (<1 ns). The high number of contacts throughout the 1 μs simulation suggest efficient interaction of both metal oxide NPs with IM (Fig. 5D). These results suggest that all NPs interacted with IM and all but AgNP could penetrate into IM. The inability of AgNP to penetrate IM despite stable interactions (Fig. 5C) could be due to the high charge density on AgNP surface. The high surface charge results in repulsion of AgNP by lipid chains thus resulting in the inability of AgNP to penetrate into IM. On the other hand, CNT was neutral while metal oxide NPs had a relatively lower surface charge density which results in much lower repulsion by the lipid tails of IM. Trajectory analysis revealed that none of the NPs formed a stable interaction with PG during 1 μs of simulation (Fig. 7). This was further corroborated by a high fluctuation in distance between NP and PG (Fig. 5E) as well as occasional contacts (Fig. 5F).

These results indicate that NPs interact with OM and IM which could potentially alter the integrity of these membranes resulting in bacterial damage. On the contrary, PG does not significantly interact with any of the investigated NPs and could possibly prevent penetration of NPs through the cell wall. This could, in turn, protect the IM from interaction, and resultant damage, from NPs. Gram-negative bacteria have a thin cell wall compared to Gram-positive bacteria hence, it is expected that PG may have a less predominant protective role in Gram-negative bacteria such *Klebsiella* used in the present study. However, Gram-negative bacteria possess OM which acts as a barrier for entry of molecules like antibacterials. MD studies revealed that OM also acts a barrier against NPs and prevented entry of all NPs tested in this study.

### Effect on biofilms

**Fig. 8:**
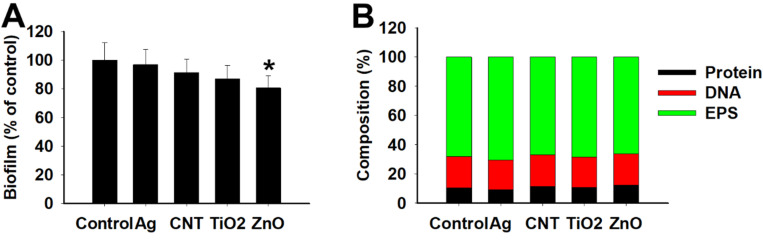
(A) Disruption of *K. quasipneumoniae* biofilm following 6 h incubation with 100 μg ml^−1^ NPs. Data is mean ± SD (*n* = 6/group). *p < 0.05 compared to control determined by One-way ANOVA and *post hoc* Tukey's test (B) *K. quasipneumoniae* biofilm composition following 72 h incubation with 100 μg ml^−1^ NPs.

Bacterial cells are embedded in the biofilm matrix and the protective layer of biofilm could potentially prevent direct NP-bacteria contact. Therefore, NPs are expected to penetrate the biofilm layer to interact with bacterial cells. NPs could potentially interact with one or more biofilm components (proteins, DNA and EPS) resulting in disruption of biofilm matrix. Therefore, the ability of NPs to disrupt mature biofilm was determined following 6 h incubation with NPs at 100 μg ml^−1^. The biofilm content estimated after 6 h was significantly (*p* < 0.05) reduced in ZnO NP-treated cells suggesting that ZnO could potentially disrupt biofilm. CNT and TiO_2_ NP also exhibited a small, but statistically insignificant (*p* > 0.05), disruption of biofilm while AgNP was practically inactive (Fig. 8A).

The interaction of NPs with biofilm was further evaluated by MD simulations. To the best of our knowledge, a computational model of biofilm has not been developed thus necessitating development of a CG model of biofilm. It may be argued that the composition of biofilm may be altered due to incubation with different NPs and hence, different models may need to be developed depending on biofilm composition. Therefore, as a first step, the relative composition of biofilm produced by control cells was compared with NP-treated cells. Quantitative analysis of biofilm extracted from control cells revealed that protein : DNA : EPS ratio by weight was approximately 1 : 2 : 6 which is comparable to that reported in clinical strains of *K. pneumoniae*.^44^ The composition of biofilm extracted from NP-treated cells was comparable to that observed in control cells (Fig. 8B) suggesting that biofilm composition was not significantly altered. Biofilm contains large number of proteins and DNA fragments with molecular weights spanning several kDa/kbp. Given the diversity in proteins and DNA, their identity is usually difficult to ascertain and is often not the subject of most of the investigations.^44^ On the other hand, EPS composition is often reported due to the relative homogeneity in composition.

EPS are an important component of biofilms and under a given set of conditions, bacteria produce predominantly one type of EPS. The amount of biofilm/EPS produced by bacteria is influenced by a variety of factors such as medium composition and stress conditions. These changes (increase or decrease) in biofilm/EPS production are often accompanied by changes in monosaccharide composition and/or chain length (molecular weight) of the EPS.^97–104^ Hence, we investigated EPS composition of the extracted EPS by FTIR, ^1^H-NMR and gel permeation chromatography (GPC) to compare the basic structure of the extracted polysaccharides. Spectroscopic (FTIR and NMR) and GPC analysis revealed that composition of EPS produced by NP-exposed cells was comparable to EPS produced by control cells (SI Fig. S8 and S9). Similarly, EPS produced by control and NP-exposed cells induced similar degree of cytotoxicity and free radical production in immune cells (macrophages) and epithelial cells (SI Fig. S10). The detailed procedure and results are provided in SI. These results suggest that NP-exposed cells produce EPS with chemical composition and biological effects comparable to that EPS produced by control cells. Hence, a single model of EPS and thus, biofilm could be used to study NP–biofilm interactions.

**Fig. 9:**
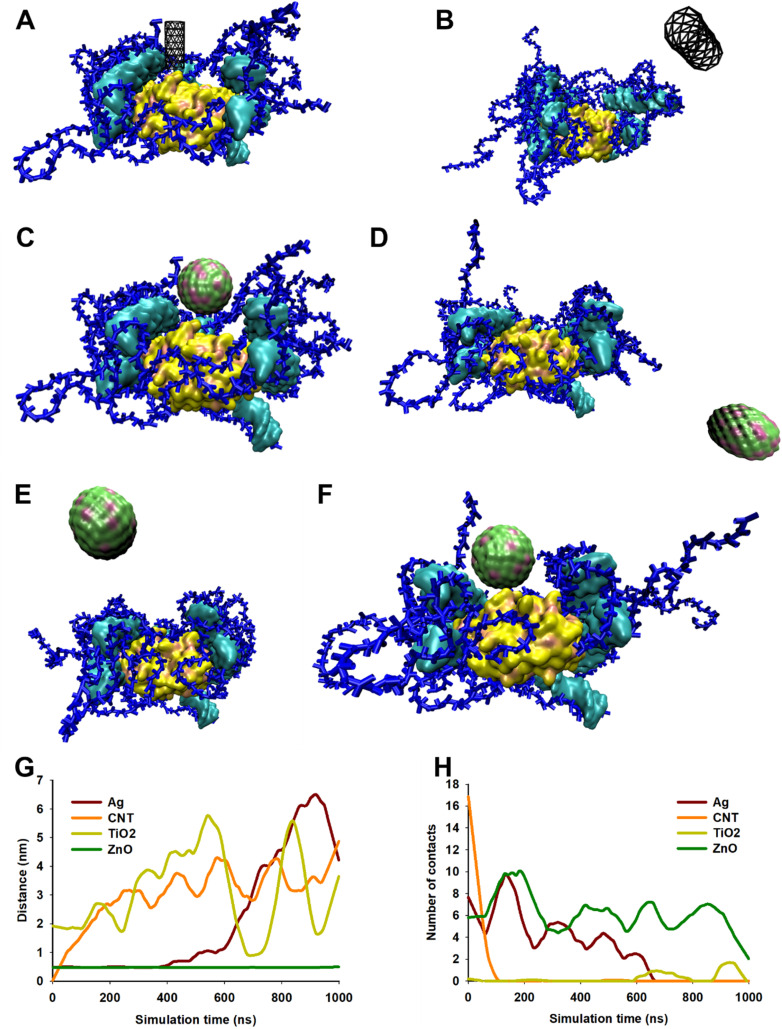
(A–F) Trajectory snapshots showing interaction between biofilm and NPs. (A) and (B) show initial (0 μs) and final (1 μs) trajectory snapshot of interaction between biofilm and CNT, respectively. (C) and (D) show initial (0 μs) and final (1 μs) trajectory snapshot of interaction between biofilm and AgNP, respectively. (E) and (F) show final (1 μs) trajectory snapshot of interaction between biofilm and TiO_2_ NP and ZnO NP, respectively. The initial position of TiO_2_ and ZnO NPs were same as that of AgNP and are not shown. The biofilm is composed of 1 molecule of TUF1 protein (yellow color), 5 molecules of 24-bp DNA (cyan color) and 15 molecules of EPS each containing 51 monosaccharide residues (blue color). Water and ions are not shown for clarity. (G) Shows distance between biofilm and NP during 1 μs of simulation. (H) Shows number of contacts between biofilm and NP during 1 μs of simulation. The lateral dimensions of box were approximately 21.6 nm × 14.8 nm.

**Fig. 10:**
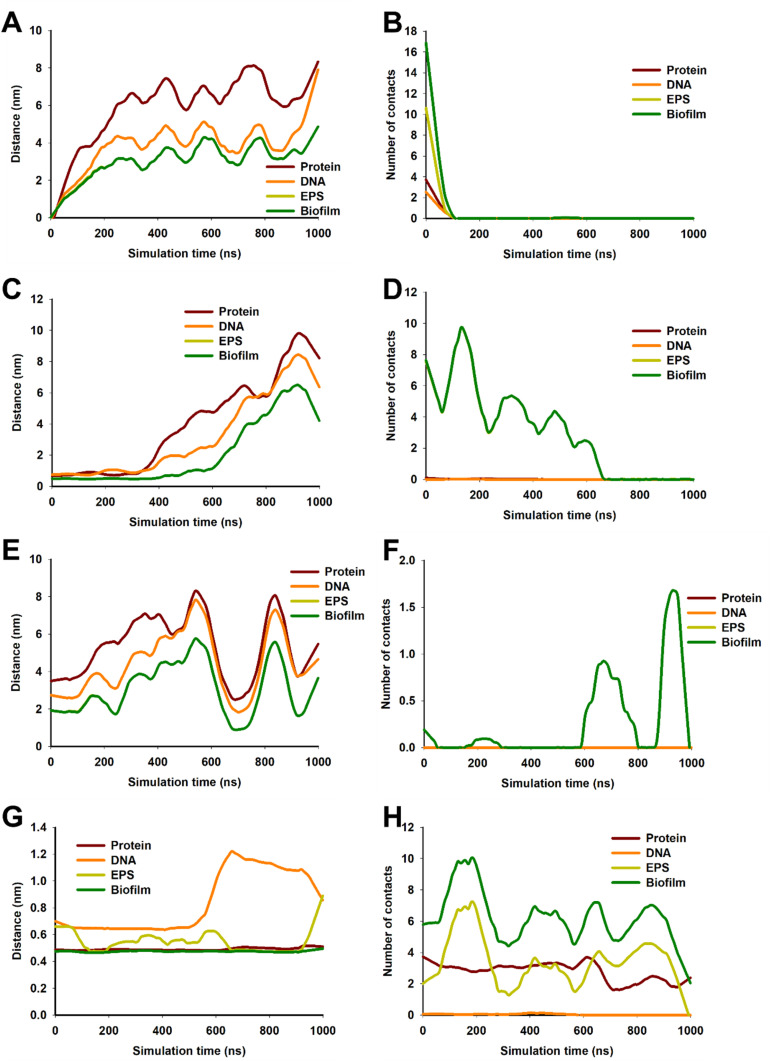
Interaction of NPs with biofilm and its components (protein, DNA and EPS) with CNT (A and B), Ag (C and D), TiO_2_ (E and F), and ZnO NP (G and H). A, C, E and G show distance between biofilm and its components and NPs during 1 μs of simulation. B, D, F and H show number of contacts between biofilm and its components and NPs during 1 μs of simulation. The line graphs of EPS and biofilm overlap in A to F.

Initial MD simulations were performed using a biofilm model mimicking the experimentally determined biofilm composition (protein : DNA : EPS in weight ratios of 1 : 2 : 6). The high EPS content in this model resulted in “burying” of protein and DNA molecules in EPS molecules hence, NPs were unable to come in contact with protein and DNA. Therefore, to study the interactions between NPs and all biofilm components, the proportion of EPS and DNA was reduced. The biofilm studied were had a composition protein : DNA : EPS 1 : 1.3 : 1.4. Trajectory analysis over 1 μs simulation revealed that CNT drifted away from biofilm (Fig. 9A and B). Similar results were also obtained with Ag and TiO_2_ NP (Fig. 9C–E) while ZnO NP remained in close proximity to the biofilm (Fig. 9F). These observations were further corroborated by distance and number of contacts measured over the entire length of simulation (Fig. 9G and H). TiO_2_ NP practically did not interact with biofilm and moved away from the biofilm during the equilibration step. At the beginning of the 1 μs production run, the distance between biofilm and TiO_2_ NP was ∼2 nm and no contacts were observed. During the course of simulation, the TiO_2_ NP moved randomly in the aqueous medium and interacted with biofilm at ∼600 ns and ∼900 ns. We believe that these were random interactions and does not signify an efficient interaction. CNT was also found to rapidly move away from biofilm and in ∼100 ns no CNT-biofilm contacts were observed. AgNP also interacted for ∼400 ns and then drifted away. Nevertheless, the interaction was relatively efficient as observed from the high number of contacts. ZnO NP appeared to stably interact with biofilm as the NP–biofilm distance remained ∼0.47 nm which is the minimum distance between two “regular” Martini beads. The number of contacts between NP and biofilm remained high enough to confirm stable NP–biofilm interaction. The biofilm is composed of protein, DNA and EPS therefore, the interactions of these components with NPs was also studied (Fig. 10). CNT did not interact with any of the three components of biofilm beyond the initial 100 ns (Fig. 10A and B). AgNP and TiO_2_ NP interacted with EPS only but did not interact with protein or DNA (Fig. 10C–F). ZnO NP interacted with protein and EPS but not DNA (Fig. 10G and H). These results suggest that ZnO NP interacted with biofilm with the highest efficiency amongst the four NPs which is further corroborated by biofilm disruption assay (Fig. 8A) where ZnO NP were found to cause maximum biofilm disruption.

The biofilm model used in the current study is not devoid of limitations. Bacterial biofilm is composed of several types of proteins, EPS, lipids and small molecules along with cells. The present model contains only DNA, protein and EPS while other important components are excluded due to the high computational cost required to run such complex systems. We believe that a more suitable model containing multiple proteins, different EPS types, small molecules, lipids and model cells present in biofilm will provide a more detailed picture of NP–biofilm interactions.

## Conclusions

The present study investigated the effect of NPs on viability and EPS production in *K. quasipneumoniae* ATCC 700603. The selected microbial strain is a biofilm-producing clinical isolate isolated from urine of a hospitalised patient^67^ and is often employed as a positive control in biofilm assays.^105^ Additionally, this strain produces ESBL and is used as a quality control strain in antibiotic susceptibility testing.^67,105^ Metallic NPs (Ag, TiO_2_ and ZnO) as well as CNT reduced cell viability and inhibited biofilm production in a concentration-dependent manner. The reduction in biofilm content was primarily attributed to a decrease in cell viability and, in case of ZnO NP, biofilm disruption. Experimental studies indicated that cell envelope was damaged as evident from OM and IM damage. CG models of all metallic NPs used in the study were developed since these are neither defined in Martini FF nor reported earlier in literature. Similarly, FF parameters (bonded terms) for cyclopropane lipids in IM were also developed. MD simulations supplemented experimental findings and revealed that all NPs interacted with OM, IM and PG to different extent. However, striking differences were observed between experimental and MD simulation results. The most notable discrepancy was that NPs showed limited interactions with OM and PG in MD simulations while experimental results showed significant damage to cell wall/membrane. This discrepancy can be due to a multitude of factors and limitations inherent in MD simulations. First, NPs can induce cell death indirectly by producing free radicals in cell or by releasing ions (*e.g.*, Ag^+^). These reactive moieties react with biomolecules and disrupt cellular homeostasis resulting in microbial killing.^106,107^ Force fields like Martini represent molecules with elastic bonds and does not support bond formation/breakage.^91^ Second, NPs can sorb organic compounds and proteins from culture medium and the NP corona can influence interactions with cells^108^ and hence, modulate cytotoxic responses. MD simulations, on the other hand, usually predict NP–cell interactions in water (with or without ions) and thus, do not accurately capture real-life conditions. Third, membranes contain large number of proteins and NPs are known to induce protein misfolding which could result in leakage in membranes. Proteins can easily unfold/misfold in Martini and the three-dimensional structure of protein is usually maintained by adding extra bonds (such as elnedyn network) or by adding constraints/restraints to the protein structure.^91^ However, these measures could also prevent misfolding of proteins on NP surface thus obscuring study of important phenomena. Fourth, MD simulations employ simple models and small timescales (up to tens of microseconds) due to high computational cost however, this simplification reduces the possibility of observing slow phenomena and rare events. A model of biofilm was also developed as the same has apparently not been reported in literature. ZnO NPs were found to interact with biofilm and corroborate experimental findings where ZnO NPs disrupted preformed biofilm. It is envisaged that future studies will employ more detailed experimental investigations like microscopic evaluations and more complex MD models for better elucidation of effects of NPs on bacterial cells and biofilms. In conclusion, experimental studies show that NPs could interact compromise integrity of OM, IM and cell wall as well as disrupt biofilms. These effects could cause bacterial damage and cell death as well as reduce biofilm production. The results of MD simulations, although helpful, need to be examined with caution due to limitations in parameterization of test systems. The strain used in the present study represents a typical case of drug-resistant bacteria due to ESBL and biofilm production. The ability of NPs to reduce cell viability and biofilm production suggest the potential of NPs to treat infection caused by multidrug resistant bacteria. It is pertinent to mention here that antimicrobial susceptibility guidelines such as those promulgated Clinical and Laboratory Standards Institute (CLSI; CLSI M26 guideline)^109^ require killing of 99.9% of bacteria to qualify as a bactericidal compound. None of the NPs could neither attain 99.9% killing nor 99.9% reduction in biofilm production even at the highest concentration thus, NPs could not be used as a standalone therapy. Since metallic NPs are used clinically, their incorporation in topical treatments could be helpful in controlling skin and wound infections along with antibiotics. Further, surface coating with these NPs could also help in control of biofilm production on abiotic surfaces such as catheters.

## Author contributions

R. P. S., T. K. and D. K. contributed to study conception and design. Material preparation, data collection and analysis were performed by A. D., N. G. and R. K., R. P. S. and T. K. carried out simulations and simulation data analysis. The first draft of the manuscript was written by A. D. and R. P. S. All authors read and approved the final manuscript.

## Conflicts of interest

There are no conflicts of interest to declare.

## Supplementary Material

RA-015-D5RA04828A-s001

## Data Availability

The python scripts used in the study are available on GitHub, the links for which have been included as part of the manuscript and supplementary information (SI). The topology files for new lipids are included as Appendix in SI. Supplementary information: additional results of MD simulations, EPS extraction and *in vitro* studies are included in supporting information. See DOI: https://doi.org/10.1039/d5ra04828a.
