# Supplementary material for: Antibacterial and antibiofilm activity of nanoparticles in Klebsiella quasipneumoniae
Source: RSC Adv. 2025 Nov 10;15(51):43576–94. doi: 10.1039/d5ra04828a (PMC12598463; doi:10.1039/d5ra04828a)
Supplement: RA-015-D5RA04828A-s001 [file RA-015-D5RA04828A-s001.pdf]

Supplementary Information (SI) for RSC Advances.
This journal is © The Royal Society of Chemistry 2025

Supplementary Information

Antibacterial and antibiofilm activity of nanoparticles in *Klebsiella quasipneumoniae*

Asha Devi,[a] Rohit Kaundal,[a] Neha Gupta,[a] Deepak Kumar,[a] Raman Preet Singh,[a,b,*] Taranpreet Kaur[c,*]

[a]School of Pharmaceutical Sciences, Shoolini University, Solan (HP), India – 173 229

[b]Department of Pharmacy, Government Polytechnic College, Patiala (PB), India – 147 001

[c]Department of Biotechnology, Government Mohindra College, Patiala (PB), India – 147 001

E-mail: ashaa3456@gmail.com (Asha Devi), rohitkaundal68@gmail.com (Rohit Kaundal), nehagupta5466@gmail.com (Neha Gupta), guptadeepak002@gmail.com (Deepak Kumar), ramanps@hotmail.com (Raman Preet Singh), taranpreet.kaur@outlook.com (Taranpreet Kaur)

* Corresponding author: Email: ramanpreetsingh@hotmail.com, tararnpreet.kaur@outlook.com; Tel.: +91-175-2321695

Supplementary Methods

Bilayer membrane simulations

The Gram-negative inner membrane (IM) composition was adapted from literature.[1] The all-atom (AA) IM, equilibrated for 1 μs, was obtained from CHARMM-GUI[1] and further equilibrated for 200 ns. The IM was symmetric (same composition in outer and inner membrane leaflets) and both leaflets contained 100 lipids each as summarized in table S1. AA IM was enclosed in a box measuring 7.98x7.98x10.00 nm^3 which also contained 76 Na^+ and 30 Cl^- ions alongwith 11550 TIP3P[2] water molecules. The AA IM was energy minimized using steepest descent algorithm (5000 steps) with position constraints applied on the phosphorus atoms. The IM were then equilibrated by stepwise releasing position restraints followed by a production run of 200 ns as described in main text for QMPE membrane. The timestep was 1 fs during equilibration runs and 2 fs during production run.

The AA IM composition was converted to coarse-grained (CG) composition as defined in the Martini framework[3, 4] using C1 bead to represent cyclopropane fatty acids and bonded parameters optimised as described below. The AA and corresponding CG membrane lipids were as described in table S1. CG topologies of POPG, POPE, DOPE and CDL2 are already defined in Martini FF and were adopted. Bonded parameters of PMPE and PMPG were optimised in this study using QMPE as model lipid as described below and the topologies are included in the Appendix.

Area per lipid (APL) and membrane thickness were calculated as described elsewhere.[1] Briefly, the box size in x and y direction were obtained for the last 100 ns (AA membranes) or 500 ns (CG membranes) of production run. The box area was obtained by multiplying box size in x and y directions and divided by number of lipid molecules per leaflet. Membrane thickness was measured as the distance between phosphate group (in AA simulations) or

phosphate beads (in CG simulations) in outer and inner leaflet of membrane using *gmx density* tool.

Table S1. Composition of membrane used in AA and Martini simulations.

AA composition		CG composition	
Component	No. of lipids per leaflet	Martini lipid	No. of lipids per leaflet
QMPE	12	PMPE	12
PMPE	46	PMPE	46
PYPG	9	POPG	9
PMPG	10	PMPG	10
POPE	13	POPE	13
OYPE	8	DOPE	8
PVCL2	2	CDL2	2
Total	100		100

The composition shown is for symmetric bilayer membrane where upper and lower leaflets contained 100 lipids each.

Optimisation of bonded parameters of cyclopropane fatty acids

An AA QMPE bilayer membrane comprising 58 QMPE lipids each in upper and lower leaflet were generated using CHARMM-GUI Membrane Builder.[5] The lipid bilayer was enclosed in a box measuring 6.01x6.01x8.50 nm3 which also contained 11 Na+ and 11 Cl- ions alongwith 4802 TIP3P water molecules. Similarly, a starting CG membrane was generated using CHARMM-GUI Martini Maker and a bilayer membrane containing 58 DPPE molecules each in upper and lower leaflet were generated. Previous studies have described the cyclopropane-containing region using Martini beadtype representing saturated fatty acid tail[6, 7] hence, C1 beadtype was employed. Thus, QMPE could be mapped to a structure

identical to DPPE but with differences in bonded parameters. The box size was 5.85x5.85x8.50 nm^3 and contained 11 Na^+ and 11 Cl^- ions alongwith 1067 CG water molecules. Simulations were run for AA and CG IM models as described above.

Citrate binding on AgNP simulations

An atomic cluster of ~2.5 nm diameter containing 531 Ag atoms (described in the main text of manuscript) was used. The Ag NP was placed in a cubic box with each side measuring 6.45 nm. Completely ionized form of citric acid molecules (-3 e charge on each citrate) was used as described elsewhere.[8] The CHARMM36[9] parameters for Ag NP and citrate were obtained using Nanomaterial Modeler[10] and Ligand Reader and Modeler[11] modules of CHARMM-GUI,[12] respectively. The simulation box containing Ag NP was then populated with 100 citrate molecules and 300 sodium ions for charge neutralization. Simulations were then performed using Gromacs software as described above with minor modifications. Ag NP was restrained in the centre of the simulation box using strong position restraints (1000 kJ/mol.nm^2 in x, y and z direction) during all simulation steps. The system was energy minimised for 5000 steps using steepest descent followed by NVT equilibration for 125,000 steps (1 fs timestep) and NVT production run for 200 ns (2 fs timestep). Leap frog algorithm was used during equilibration and production runs. All atoms were coupled to temperature bath and maintained at 310K using velocity rescaling and Verlet cut-off scheme was used as described above. Hydrogen bonds were constrained using LINCS algorithm.[13] The equilibrated structure obtained from this 200 ns simulation in vacuum was then solvated with 7646 TIP3P water.[2] The system was again subject to minimization, equilibration and production runs as described for vacuum simulations with the exception that a 200 ns NPT production run was performed using Parrinello-Rahman barostat. The number of citrate molecules within 0.5 nm of Ag NP surface was determined during the 200 ns production runs in vacuum and in water using in-built tools available in Gromacs (*gmx select*). Additionally,

radial distribution function (RDF) values were determined for various heavy (non-hydrogen) atoms of citrate molecules bound to Ag NP surface. RDF values were determined for the last 100 ns of simulation using *gmx rdf* tool available in Gromacs.

Contact angle simulations

We constructed a single layer slab of beads separated by 0.47 nm (default value in Martini FF) measuring ~19 nm x 20 nm using an in-house python script developed for building CG graphene (https://github.com/ramanpsingh/CGGrapheneBuilder). A sphere of water containing 900 regular water (W beads) and 100 anti-freeze water (WF beads) was packed in a sphere using Packmol[14] and placed on the slab. The system was minimized and equilibrated as described for membranes above with slight modifications as follows. All equilibration runs were performed with NVT ensemble for 1 ns. The NVT production run was performed for 1μs. Position restraints (1000 kJ/mol.nm^2) were applied on the slab throughout the simulation and temperature was 298K. Martini beads of increasing hydrophobicity were employed (N0, C5, C3, C2 and C1) to describe the slab and contact angle of the sphere was determined.

EPS extraction and characterization

EPS extraction was performed as described elsewhere with minor modifications.[15] Briefly, the inoculum was prepared using overnight colonies from nutrient agar as described in main manuscript. The inoculum (1 ml; OD_{600} 0.6-0.8) was transferred to 100 ml Luria broth containing 50 μg/ml NPs and incubated for 72 h in conical flasks for development of biofilm. Control cells were incubated in Luria broth for 72 h without NPs. Thereafter, 600 μl of 36.5% formaldehyde was added to each flask, kept at room temperature (1 h) with shaking (100 rpm). Then, 40 ml of 1M NaOH was added to each flask and kept at room temperature (3 h) with shaking (100 rpm). The suspension was centrifuged (20,000g, 30 min, 4ºC), supernatant was collected and dialyzed with a 12 kDa cut-off membrane for 24 h at 4ºC. The proteins and

nucleic acids were removed by addition of trichloroacetic acid (20% w/v) followed by centrifugation (20,000g, 30 min, 4ºC) after 30 minutes. Then, supernatant was mixed with 1.5 volumes of 95% ethanol and maintained at -20ºC for 24 h followed by centrifugation (20,000g, 30 min, 4ºC). The precipitated polysaccharides were collected, resuspended in water, dialyzed (12 kDa cut-off membrane) for 24 h at 4ºC and then lyophilized. The yield of polysaccharide was 2.5-4 mg per 100 ml of broth culture.

The lyophilized powder was characterized by attenuated total reflection-Fourier transform infrared (ATR-FTIR) and nuclear magnetic resonance (NMR) spectroscopy. A small amount of the lyophilized powder was placed on the diamond crystal and FTIR scans at 8 cm^{-1} resolution and 60 scans were recorded on Agilent Cary 660 FTIR.[16, 17] The polysaccharide was dissolved in deuterated water and subjected to ^{1}H-NMR analysis using Jeol JNM ECX-500 NMR at ambient temperature (20±2°C). The monosaccharides were identified using CASPER (http://www.casper.organ.su.se/casper/).[18-20] Molecular weight of polysaccharide was determined by gel permeation chromatography (GPC) as described earlier.[21]

In vitro studies

Human lung epithelial (A549) and mouse macrophage (RAW 264.7) cell lines were obtained from National Centre for Cell Sciences, Pune, India. Cell lines were maintained in culture medium (DMEM containing 10% FBS) as described earlier.[22] Cells were incubated with 100 µg/ml EPS in 96-well tissue culture plates (10,000 cells/well/200 µl) for 24 h. Thereafter, cell viability was determined by MTT as described earlier.[23] Reactive oxygen species (ROS) production in cells and nitrite release in culture supernatant were determined using dichlorofluorescein diacetate (DCFDA) and Griess reagent, respectively.[24]

Results

Optimisation of membrane parameters

Gram-negative bacteria contain cyclopropane fatty acids which are not parameterized in the standard Martini FF thus, we sought to parameterize bonded parameters cyclopropane fatty acids. QMPE was chosen as the representative AA lipid and PMPE as its CG counterpart. Martini beads were mapped on the cyclopropane fatty acid in AA QMPE model and bonded parameters for CG model were optimised. The fatty acid was mapped to a 4 bead model where the linear chain consisted of C1A-C2A-C3A-C4A. Bead C3A corresponded to cyclopropane-containing region of the fatty acid. All other beads were mapped as described in the Martini FF and shown in figure S1. The fitting of bond length and bond angle distribution of optimised parameters is shown in supporting figure S2 and summarised in table S2. APL of AA and CG membrane were comparable (0.62±0.01 nm^2 and 0.60±0.01 nm^2, respectively) as well as membrane thickness (Figure S3A) suggesting relevance of the optimised bonded parameters. APL of AA IM was 0.68±0.01 nm^2 compared to 0.62±0.01 nm^2 in CG IM model generated using composition in table S1. The membrane thickness in AA and CG IM models was also comparable (Figure S3B).

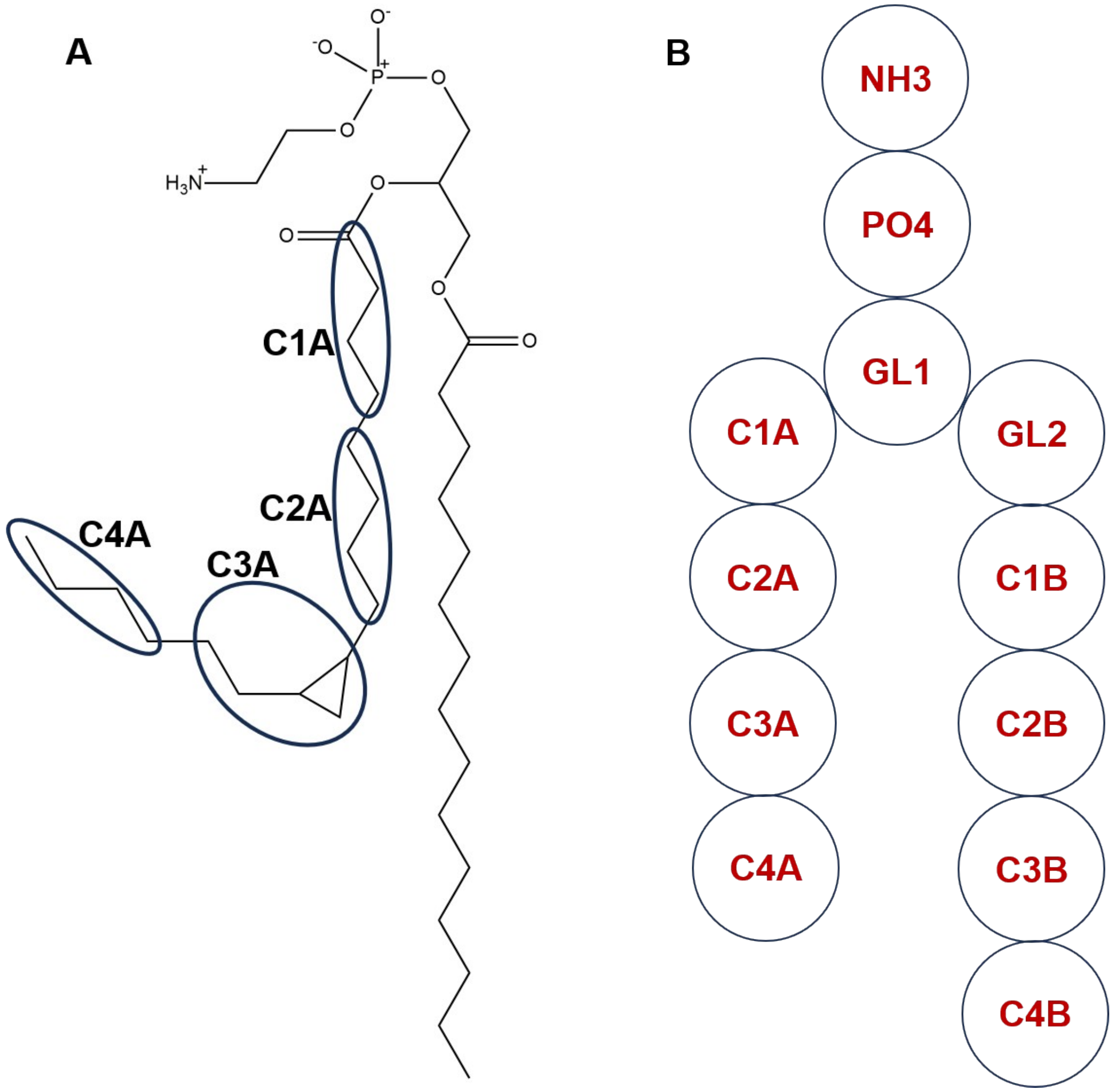

Figure S1. Mapping of Martini beads on QMPE. (A) shows the mapping of beads on cyclopropane fatty acid chain. The remaining atoms were mapped onto Martini beads using standard Martini conventions. (B) shows CG model of QMPE.

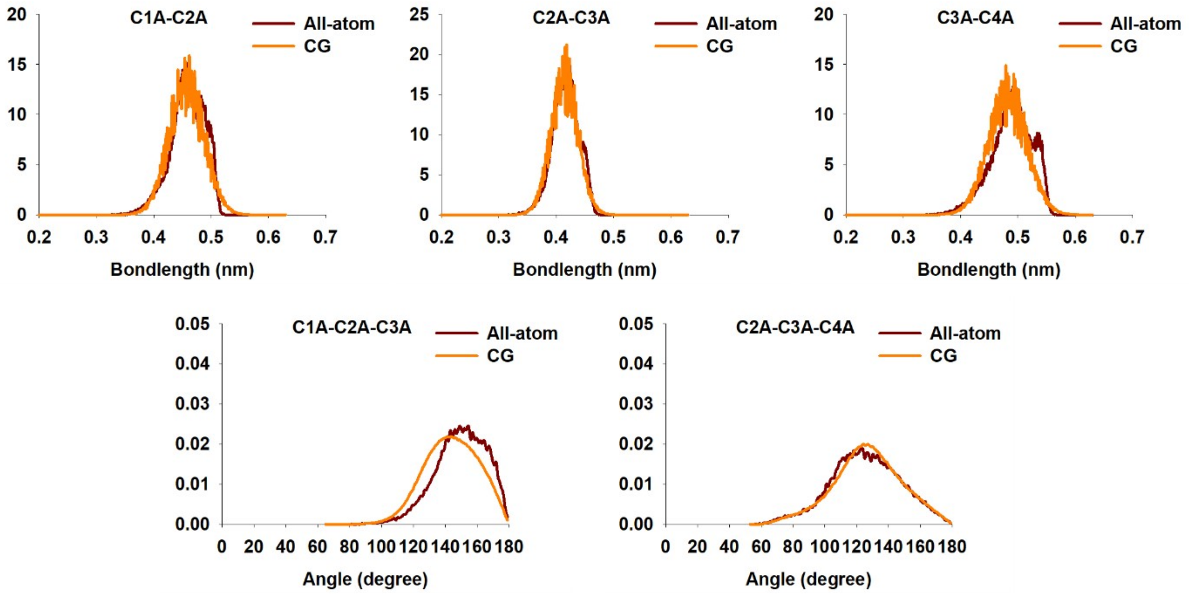

Figure S2. Fitted bonded parameters for cyclopropane fatty acid beads. Bead names are the same as in figure S1.

Table S2. Optimised bonded parameters of cyclopropane fatty acid in QMPE.

**Bonds**	**b_0 (nm)** **k_b (kJ.mol^{-1}.nm^{-2})**	**Angles**	**θ_0 (degree)** **k_θ (kJ.mol^{-1}.rad^{-2})**
C1A-C2A	0.465 3000.0	C1A-C2A-C3A	180.0 30.0
C2A-C3A	0.42 5000.0	C2A-C3A-C4A	120.0 20.0
C3A-C4A	0.49 2500.0		

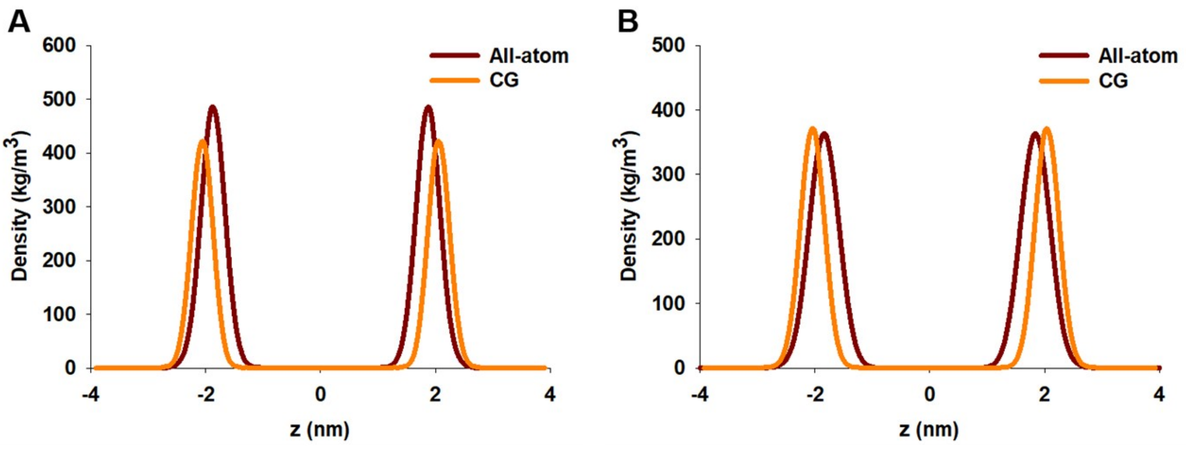

Figure S3. Comparison of membrane thickness of AA and CG models. (A) shows data for membrane containing 58 QMPE (AA) and 58 PMPE (CG) molecules in inner and outer leaflet each. (B) shows comparison of AA IM and CG IM models. The CG model was generated using parameters optimised in (A) and listed in table S2. The composition of AA and CG models is shown in table S1.

Citrate binding on Ag NP surface

As observed in figure S4, 45 citrate molecules were stably bound to Ag NP surface. On the other hand, desorption of citrate happened during the initial 50 ns in water resulting in ~30 bound molecules. Analysis of the last 100 ns trajectory revealed that 45 citrate molecules (mean = 45.1, SD = 0.5; mode = 45) were stably attached in vacuum while 31 citrate molecules (mean = 31.4, SD = 1.8; mode = 31) were stably attached in water. The frequency of number of bound citrate molecules in vacuum and in water is shown in figure S5. Based on the simulation in water, the surface coverage of citrate was found to be 1.5 molecules/nm^2 which is close that observed in gold NPs (1.55 molecules/nm^2 for fully deprotonated citrate).[25] Hence, the model of gold NP described elsewhere[25] was employed in this study.

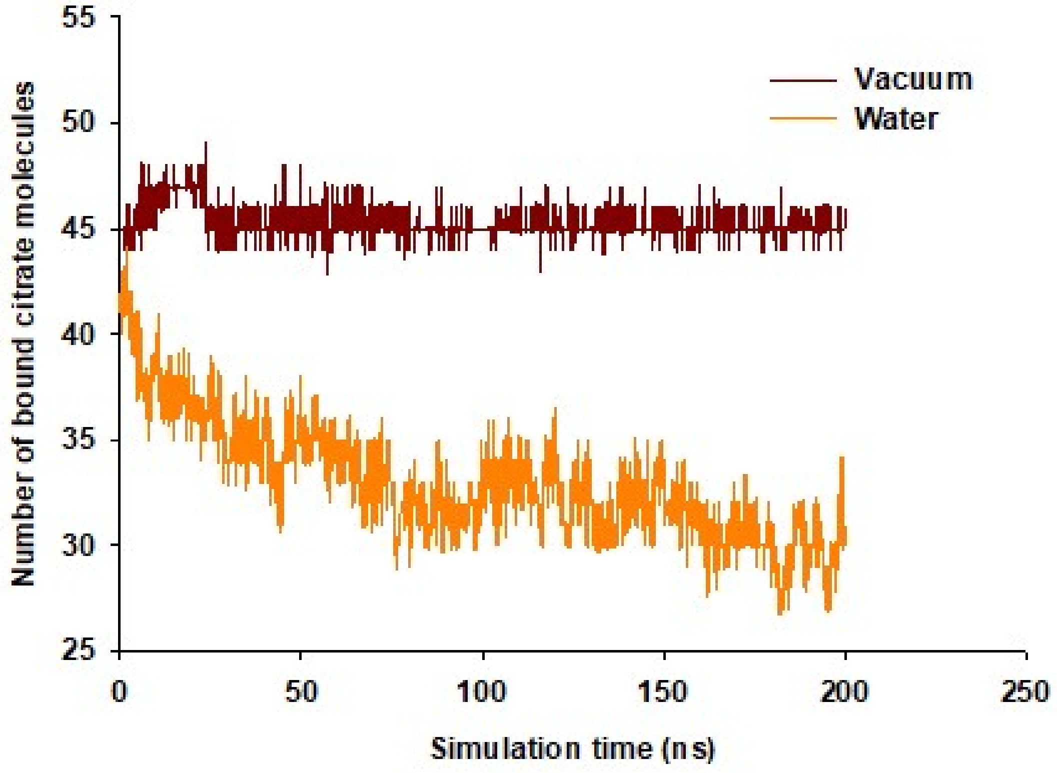

Figure S4. Number of citrate molecules bound to Ag NP surface during 200 ns simulation in vacuum and in water.

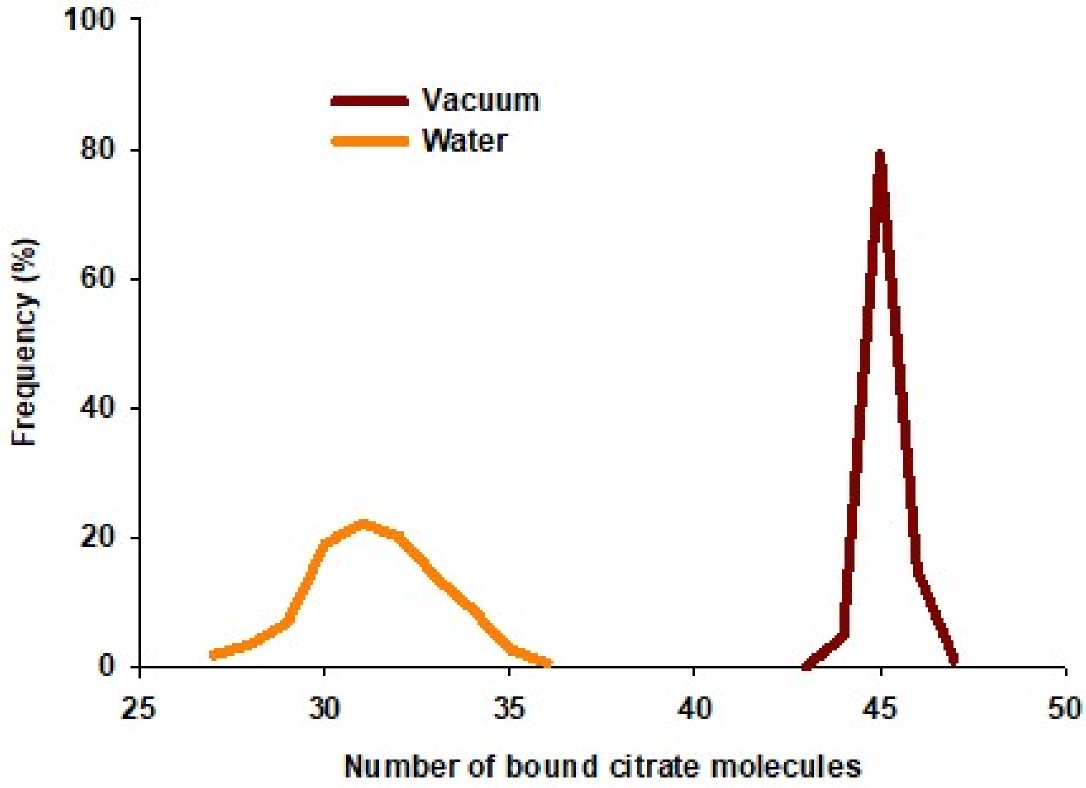

Figure S5. Frequency distribution of number of citrate molecules bound to Ag NP during last 100 ns of the total 200 ns simulation in vacuum and in water.

The mechanism of interaction was determined by measuring the number of different atoms present in vicinity (with 0.5 nm) of Ag NP surface. Due to deprotonation, the charge distribution on both oxygens in a carboxylic group is similar. Further, the terminal carboxyl groups are chemically equivalent hence, the oxygen and carbon atoms on -COO^- on both termini were combined for analysis and are depicted as **1** and **3**, respectively, in figure S6A. The oxygen and carbon atoms on central COO^- are depicted separately as **2** and **4**, respectively. The oxygen on hydroxyl group is depicted as **7**. The carbon atoms attached to terminal and central COO^- are depicted as **5** and **6**, respectively.

The binding fraction for a group of atoms was determined using the formula $\frac{Number\ of\ atoms}{Number\ of\ citrate\ molecules}$. This formula helps in finding the fraction of atoms in vicinity of Ag NP. For example, **1** is composed of group of 4 equivalent O atoms and if all the 4 atoms are bound to Ag NP, the binding fraction will be 4. A lower value of binding indicates relatively lesser number of terminal carboxyl O attached to Ag NP surface. The binding fraction for all heavy atoms in vacuum and in water are shown in figures S6B and S6C, respectively and corresponding RDF shown in figures S6D and S6E. The data averaged over the entire simulation (200 ns) and the last 100 ns simulation in vacuum and water is summarized in table S3. RDF analysis over the last 100 ns of simulation revealed that the carboxyl O **1** and **2** and the hydroxyl O **7** were the closest to Ag NP followed by **3** and **5** in vacuum (Figure S6D). On the other hand, **1** and **7** were closest to surface alongwith **3** and **5** in water while **2** showed interaction to a small extent (Figure S6E). These results suggest that the interaction of citrate with Ag NP surface occurs via carboxyl and hydroxyl groups.

Table S3. Binding fraction of heavy (non-hydrogen) atoms in citrate molecule determined in vacuum and in water during the entire length of simulation (200 ns) or the last 100 ns of the simulation.

		Observed binding			
Atom type	Ideal binding	Vacuum		Water	
		200 ns	Last 100 ns	200 ns	Last 100 ns
1	4	2.3±0.1	2.3±0.1	3.0±0.2	3.1±0.2
2	2	1.1±0.0	1.1±0.0	1.0±0.1	1.0±0.1
3	2	1.2±0.0	1.2±0.0	1.6±0.1	1.7±0.1
4	2	0.6±0.0	0.6±0.0	0.6±0.1	0.7±0.1
5	2	1.3±0.0	1.3±0.0	1.7±0.1	1.8±0.1
6	1	0.7±0.0	0.7±0.0	0.9±0.1	0.9±0.0
7	1	0.6±0.0	0.6±0.0	0.8±0.1	0.8±0.1

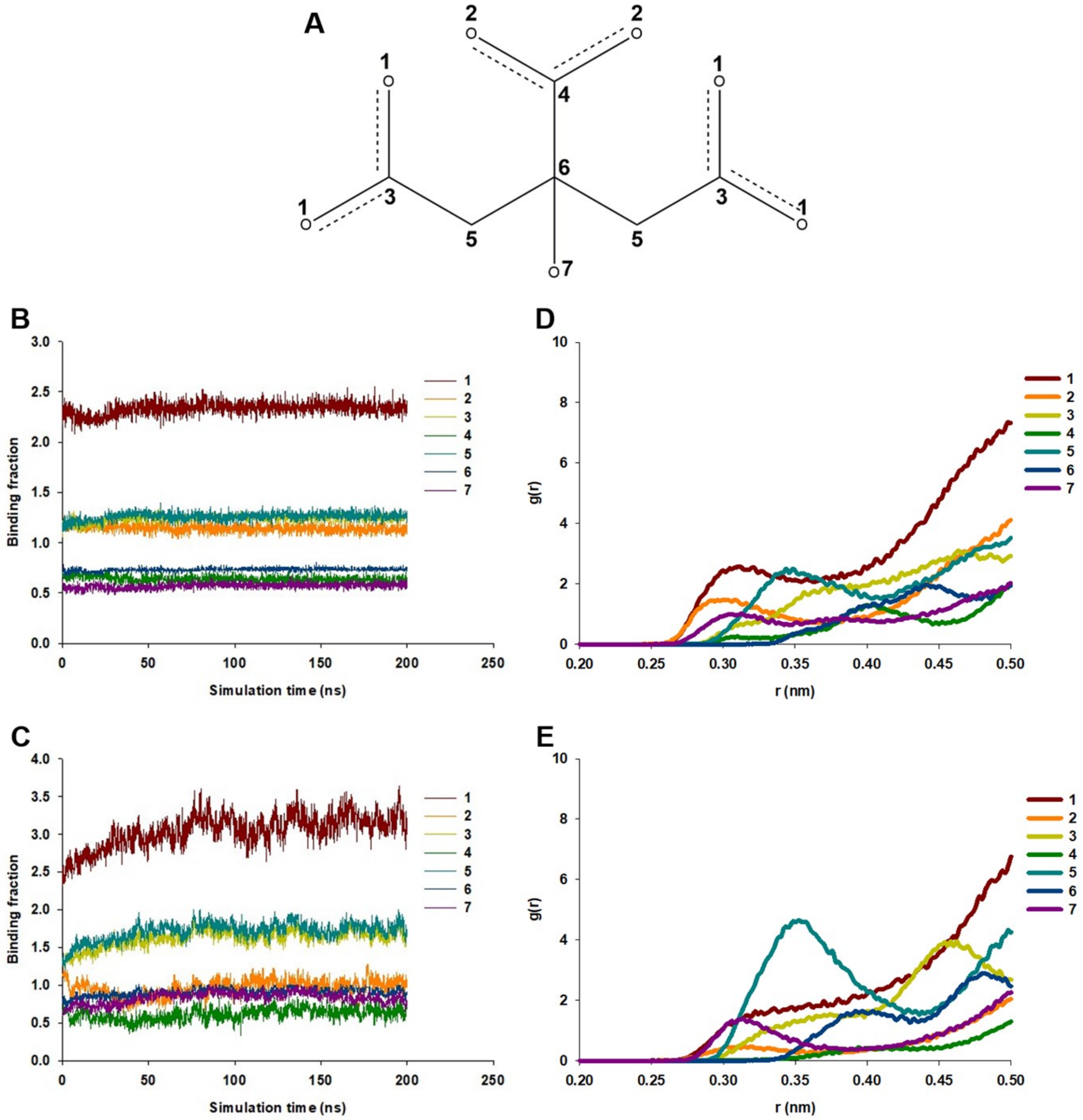

Figure S6. (A) shows labelling of heavy (non-hydrogen) atoms in citrate molecule. (B) and (C) show binding fraction of heavy (non-hydrogen) atoms in citrate molecule determined in vacuum and in water, respectively, during the entire length of simulation (200 ns). (D) and (E) show RDF plots of heavy (non-hydrogen) atoms in citrate molecule determined in vacuum and in water, respectively, for the last 100 ns of the total 200 ns simulation.

Bead types for Ag, TiO_2 and ZnO NPs

The slab was assigned different beadtypes and contact angle of a water droplet (containing 1000 CG water beads) was determined after 1μs simulation. When N0, C5, C3, C2 and C1 beads were assigned to atoms of the slab, water formed a droplet on the slab with contact angles ~40°, ~45°, ~50°, ~70° and ~80°, respectively, on the slab (Figure S7). It is worth mentioning here that the contact angle on N0 slab was not consistent: in 2 simulation replicates, water beads spread on the surface while in 4 other replicates, contact angle was in the range of 30-40° and thus the latter was accepted as the more reliable value. Water contact angle of 61.3 and 73° have been reported for TiO_2 surfaces[26, 27] while contact angle of 81° have been reported on ZnO surface.[28] Since the contact angle formed was close to experimental values, C2 beads were assigned to TiO_2 NPs and C1 beads to ZnO NPs. A water contact angle of 64° has been reported for silver metal[29] thus, bead type C2 was assigned to Ag NP.

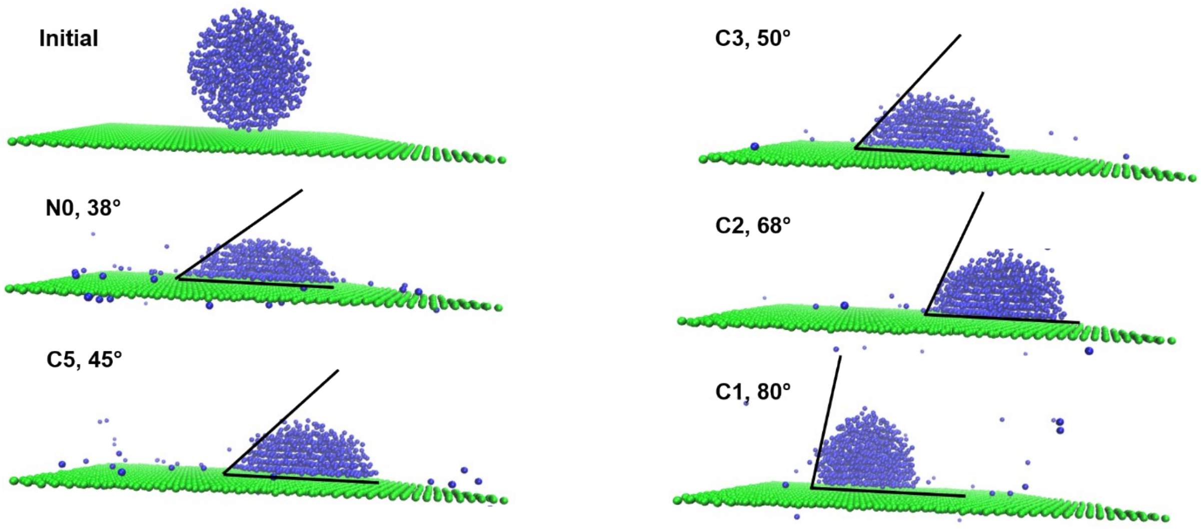

Figure S7. Contact angle of a water droplet on slabs with different beadtypes. The initial configuration was same in all simulations and differed only in beadtypes assigned to the slab

(N0, C5, C3, C2 and C1). The initial water droplet contained 1000 CG water beads and each simulation was run for 1µs.

Characterization of EPS

FTIR and ^{1}H-NMR were. FTIR spectra of EPS extracted from control cells (Figure S9) exhibited several peaks corresponding to mannans (779, 866 and 916 cm^{-1}), glucans (725, 779 and 866 cm^{-1}) and glucose (964, 1028 and 1052 cm^{-1}) reported earlier in *K. quasipneumoniae* biofilms.[30] Additionally, peaks corresponding to galactose (820 and 850 cm^{-1}) were also observed. The band at 3000-3600 cm^{-1} was due to O-H stretching vibrations and the peak centred at 2930 cm^{-1} was due to C-H stretching vibrations.[31, 32] The peaks at 1458 and 1637 cm^{-1} were due to symmetrical and asymmetrical carboxylate stretching vibrations. The peak at 1532 cm^{-1} was amide II band. The peak at 1235 cm^{-1} indicated presence of acetyl groups.[30] EPS extracted from NP-treated cells exhibited IR spectral features similar to those observed in control cells.

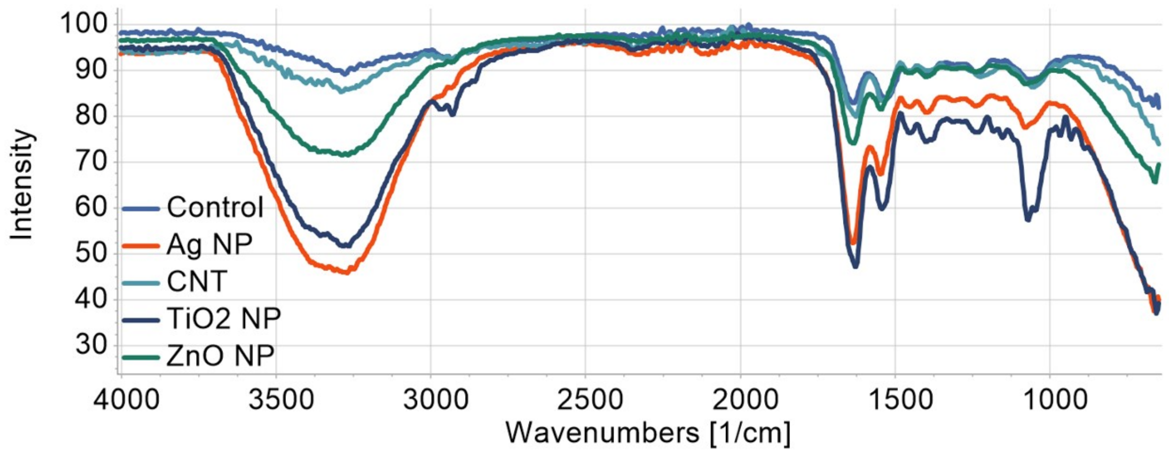

Figure S8. ATR-FTIR spectrum of *K. quasipneumoniae* EPS following 72 h incubation with 100 µg/ml NPs.

^{1}H-NMR of EPS extracted from control cells and cells exposed to different NPs was comparable with minor differences (Figure S10) suggesting that the basic structure of polysaccharide remained unchanged due to NP exposure. Unlike FTIR, ^{1}H-NMR spectra of polysaccharides are difficult to interpret because of clustering of peaks of different monosaccharides in a very small region. Hence, CASPER program was used to identify EPS composition. CASPER analysis showed that galactose, glucose, mannose and rhamnose were the major monosaccharides present in EPS from control and NP-exposed cells which is in agreement with FTIR described above as well as with previously reported polysaccharide composition of *K. quasipneumoniae* ATCC 700603.[15] It is worth mentioning here that in other strains of this organism, mannose was found to be the major monosaccharide along with galactose and glucose.[30, 33-35] We found that size separation of EPS by GPC resulted in elution of the polysaccharide as a single peak in control and NP-exposed cells. Further, the molecular weight of EPS obtained from NP-exposed cells was comparable to EPS obtained from control cells and the average molecular weight was >25 kDa (~28-35 kDa) in all cases. Together, these results suggest that NP treatment did not significantly alter the composition of EPS and thus, points towards the possibility that the observed reduction in biofilm production was due to reduced EPS production by the bacterium with a concomitant reduction in other biofilm components.

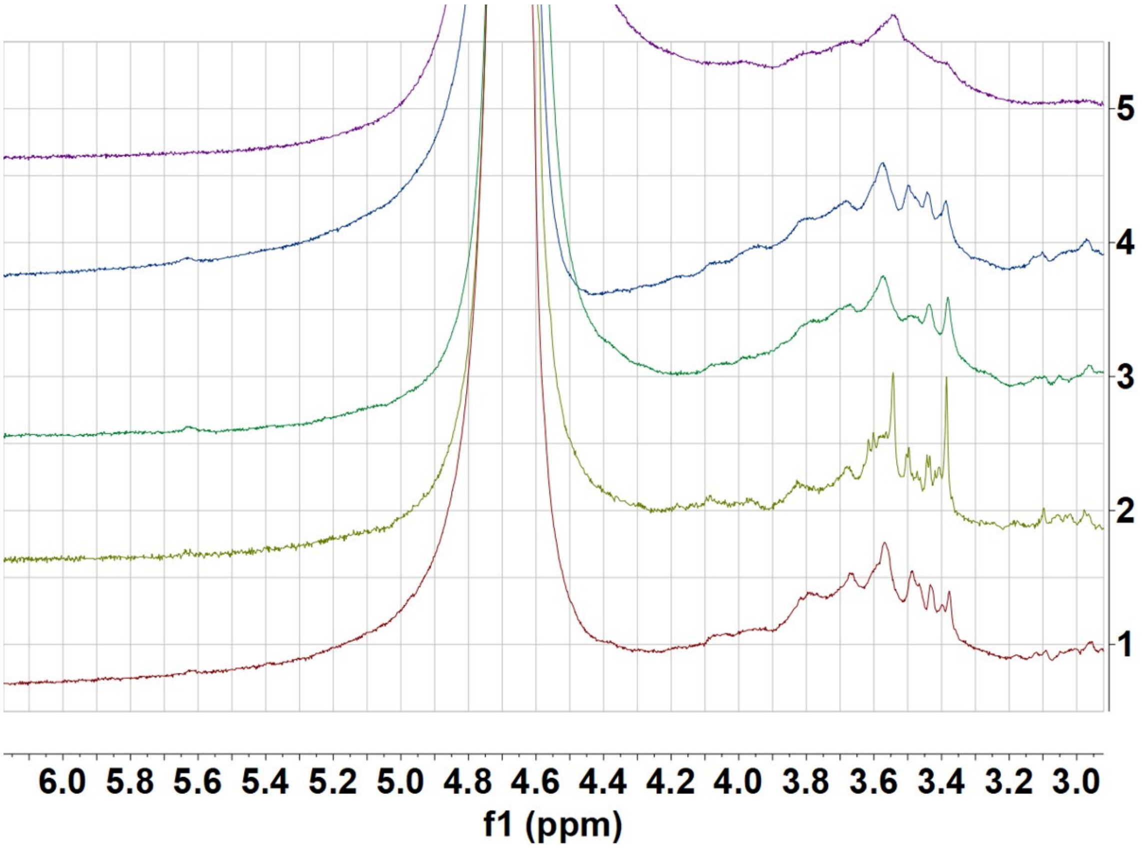

Figure S9. ^{1}H-NMR spectrum of *K. quasipneumoniae* EPS following 72 h incubation with 100 µg/ml NPs. The figure shows an overlay of EPS extracted from control cells (1) and cells treated with Ag NP (2), CNT (3), TiO_2 NP (4) and ZnO NP (5).

The analytical and spectroscopic methods of EPS characterization offer insights in EPS composition but may fail to detect subtle changes in EPS composition. There exists a possibility that these subtle changes can influence interactions between host cells and EPS. Therefore, the effect of EPS on cell viability was determined in two cell lines. Since *K. quasipneumoniae* causes lung infections, a lung epithelial cell line (A549) was included. Macrophages are professional phagocytes and play an important role in host immunity against invading pathogens hence, a macrophage cell line (RAW 264.7) was included. Cell lines were incubated with 100 µg/ml EPS and cell viability was determined after 24 h. EPS

extracted from untreated or NP-treated *K. quasipneumoniae* did not significantly (p>0.05) alter cell viability in any of the target cells (Figure S11A).

Macrophages are activated after encountering microbes/microbial products and increased free radical production is one of the hallmarks of macrophage activation (often dubbed as respiratory burst). In addition to reactive oxygen species, activated macrophages also produce reactive nitrogen species (RNS) derived from nitric oxide. RNS are highly reactive (and unstable) and produce nitrite as a stable product. EPS-stimulated nitrite production was thus used as a surrogate to determine stimulation of RNS production in macrophages. ROS and RNS production was determined after 24 h of incubation with EPS. ROS production was normalized to unstimulated cells (incubated in culture medium without EPS and referred as negative control). ROS levels were significantly increased in RAW 264.7 macrophage cells incubated with EPS extracted from untreated (control) and NP-treated bacterial cells compared to the negative control while ROS production in A549 epithelial cells was comparable to negative control (Figure S11B). Further, stimulation of ROS production in A549 cells by EPS extracted from NP-treated bacterial cells was comparable to EPS extracted from untreated bacterial cells (p>0.05). Similarly, nitrite production was elevated in EPS-treated RAW 264.7 cells and the increase in nitrite production was comparable amongst different EPS (Figure S11C). As expected, nitrite production was undetectable in A549 cells since their physiological role precludes the need to produce significantly high amounts of RNS. Therefore, it may be concluded that incubation of NP with the bacterium does not alter the biological properties of EPS studied in the present study (Figure S11).

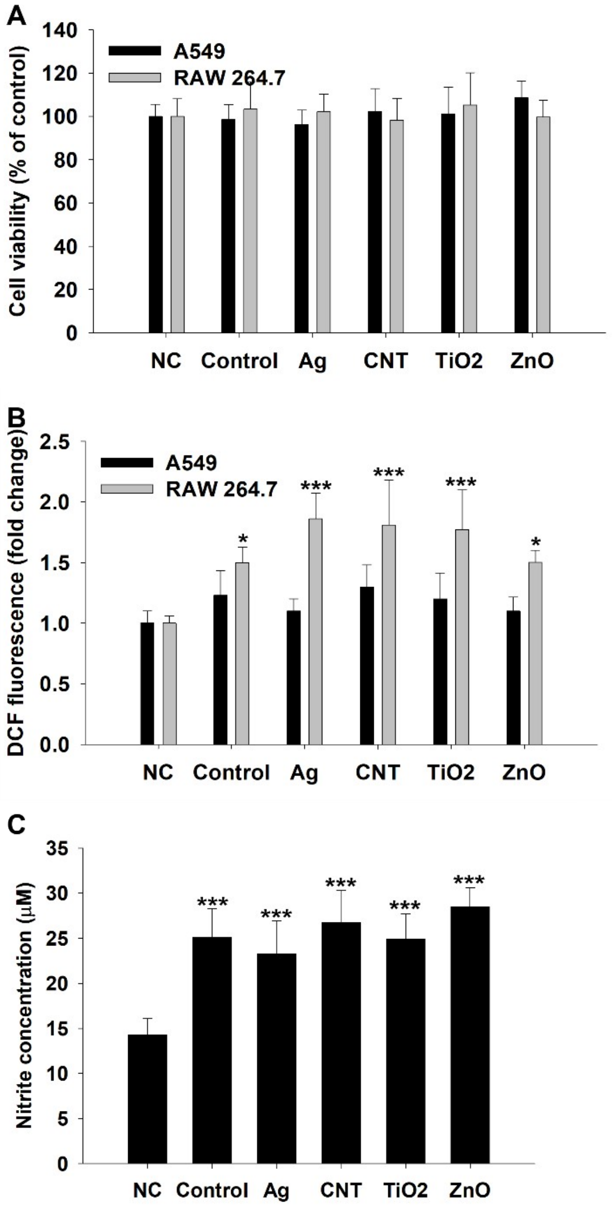

Figure S10. Effect of EPS on cell viability and free radical production in A549 and RAW 264.7 cells following 24 h incubation. (A) and (B) show cell viability and ROS production determined by MTT and DCF assay, respectively. Data is normalized to cell viability/DCF fluorescence of negative control (NC). (C) shows nitrite concentration in RAW 264.7 cell supernatants.

Data is mean±SD (n=5/group).

*p<0.05, ***p<0.001 compared to NC determined by One-way ANOVA and *post hoc* Tukey's test

References

1. I. D. Pogozheva, G. A. Armstrong, L. Kong, T. J. Hartnagel, C. A. Carpino, S. E. Gee, D. M. Picarello, A. S. Rubin, J. Lee, S. Park, A. L. Lomize and W. Im, *J Chem Inf Model*, 2022, **62**, 1036-1051.https://doi.org/10.1021/acs.jcim.1c01514
2. W. L. Jorgensen, J. Chandrasekhar, J. D. Madura, R. W. Impey and M. L. Klein, *The Journal of Chemical Physics*, 1983, **79**, 926-935.https://doi.org/10.1063/1.445869
3. S. J. Marrink, H. J. Risselada, S. Yefimov, D. P. Tieleman and A. H. de Vries, *The Journal of Physical Chemistry B*, 2007, **111**, 7812-7824.https://doi.org/10.1021/jp071097f
4. T. A. Wassenaar, H. I. Ingólfsson, R. A. Böckmann, D. P. Tieleman and S. J. Marrink, *Journal of Chemical Theory and Computation*, 2015, **11**, 2144-2155.https://doi.org/10.1021/acs.jctc.5b00209
5. E. L. Wu, X. Cheng, S. Jo, H. Rui, K. C. Song, E. M. Dávila-Contreras, Y. Qi, J. Lee, V. Monje-Galvan, R. M. Venable, J. B. Klauda and W. Im, *Journal of Computational Chemistry*, 2014, **35**, 1997-2004.https://doi.org/10.1002/jcc.23702
6. A. Maiti, A. Kumar and S. Daschakraborty, *The Journal of Physical Chemistry B*, 2023, **127**, 1607-1617.https://doi.org/10.1021/acs.jpcb.3c00541
7. K. Pluhackova and A. Horner, *BMC Biology*, 2021, **19**, 4.https://doi.org/10.1186/s12915-020-00936-8
8. G. Chong and R. Hernandez, *The Journal of Physical Chemistry C*, 2018, **122**, 19962-19969.https://doi.org/10.1021/acs.jpcc.8b05202
9. J. Huang and A. D. MacKerell, Jr., *J Comput Chem*, 2013, **34**, 2135-2145.https://doi.org/10.1002/jcc.23354

10. Y. K. Choi, N. R. Kern, S. Kim, K. Kanhaiya, Y. Afshar, S. H. Jeon, S. Jo, B. R. Brooks, J. Lee, E. B. Tadmor, H. Heinz and W. Im, *Journal of Chemical Theory and Computation*, 2022, **18**, 479-493.https://doi.org/10.1021/acs.jctc.1c00996

11. S. Kim, J. Lee, S. Jo, C. L. Brooks Iii, H. S. Lee and W. Im, *Journal of Computational Chemistry*, 2017, **38**, 1879-1886.https://doi.org/10.1002/jcc.24829

12. S. Jo, T. Kim, V. G. Iyer and W. Im, *Journal of Computational Chemistry*, 2008, **29**, 1859-1865.https://doi.org/10.1002/jcc.20945

13. B. Hess, H. Bekker, H. J. C. Berendsen and J. G. E. M. Fraaije, *Journal of Computational Chemistry*, 1997, **18**, 1463-1472.https://doi.org/10.1002/(SICI)1096-987X(199709)18:12<1463::AID-JCC4>3.0.CO;2-H

14. L. Martinez, R. Andrade, E. G. Birgin and J. M. Martinez, *J Comput Chem*, 2009, **30**, 2157-2164.https://www.ncbi.nlm.nih.gov/pubmed/19229944

15. P. M. Bales, E. M. Renke, S. L. May, Y. Shen and D. C. Nelson, *PLoS One*, 2013, **8**, e67950.https://doi.org/10.1371/journal.pone.0067950

16. A. Bisht, M. Zuñiga-Bustos, G. Prasher, S. Gautam, H. Poblete and R. P. Singh, *Langmuir*, 2022, **38**, 10173-10182.https://doi.org/10.1021/acs.langmuir.2c01190

17. S. Kumar, M. Das, R. P. Singh, S. Datir, D. S. Chauhan and S. Jain, *Colloids Surf. A Physicochem. Eng. Asp.*, 2013, **419**, 156-165.https://doi.org/10.1016/j.colsurfa.2012.11.070

18. A. Furevi, A. Ruda, T. Angles d'Ortoli, H. Mobarak, J. Stahle, C. Hamark, C. Fontana, O. Engstrom, P. Apostolica and G. Widmalm, *Carbohydr. Res.*, 2022, **513**, 108528.https://doi.org/10.1016/j.carres.2022.108528

19. P. E. Jansson, R. Stenutz and G. Widmalm, *Carbohydr. Res.*, 2006, **341**, 1003-1010.https://doi.org/10.1016/j.carres.2006.02.034

20. M. Lundborg and G. Widmalm, *Methods Mol. Biol.*, 2015, **1273**, 29-40.https://doi.org/10.1007/978-1-4939-2343-4_3

21. T. Kaur and M. Ghosh, *J. Water Health*, 2016, **15**, 103-111.https://doi.org/10.2166/wh.2016.176

22. R. P. Singh, S. S. Jhamb and P. P. Singh, *Life Sci.*, 2008, **82**, 308-314.https://doi.org/10.1016/j.lfs.2007.11.024

23. L. Mehta, S. Kumari and R. P. Singh, *Chem. Res. Toxicol.*, 2020, **33**, 1206-1214.https://doi.org/10.1021/acs.chemrestox.9b00370

24. R. P. Singh, S. Jain and P. Ramarao, *J. Nanopart. Res.*, 2013, **15**, 1985.https://doi.org/10.1007/s11051-013-1985-7

25. S. Franco-Ulloa, G. Tatulli, S. L. Bore, M. Moglianetti, P. P. Pompa, M. Cascella and M. De Vivo, *Nature Communications*, 2020, **11**, 5422.https://doi.org/10.1038/s41467-020-19164-3

26. J. S. Hwang, J. Lee and Y. H. Chang, *Macromolecular Research*, 2005, **13**, 409-417.https://doi.org/10.1007/BF03218474

27. J. H. Park and N. R. Aluru, *Molecular Simulation*, 2009, **35**, 31-37.https://doi.org/10.1080/08927020802398884

28. L. D. Trino, L. F. G. Dias, L. G. S. Albano, E. S. Bronze-Uhle, E. C. Rangel, C. F. O. Graeff and P. N. Lisboa-Filho, *Ceramics International*, 2018, **44**, 4000-4008.https://www.sciencedirect.com/science/article/pii/S0272884217326640

29. L. Somlyai-Sipos and P. Baumli, *Journal*, 2022, **12**, 1274.https://doi.org/10.3390/met12081274

30. A. K. Singh, S. Yadav, B. S. Chauhan, N. Nandy, R. Singh, K. Neogi, J. K. Roy, S. Srikrishna, R. K. Singh and P. Prakash, *Front. Microbiol.*, 2019, **10**, 669.https://doi.org/10.3389/fmicb.2019.00669

31. T. Kaur and M. Ghosh, *Carbohydr. Polym.*, 2015, **132**, 72-79.https://doi.org/10.1016/j.carbpol.2015.06.002

32. T. Kaur, J. Sharma, A. Ganguli and M. Ghosh, *Compos. Interfaces*, 2014, **21**, 143-151.https://doi.org/10.1080/15685543.2013.842116

33. P. Cescutti, G. De Benedetto and R. Rizzo, *Carbohydr. Res.*, 2016, **430**, 29-35.https://doi.org/10.1016/j.carres.2016.05.001

34. A. K. Mandal, I. K. Sen, P. Maity, S. Chattopadhyay, R. Chakraborty, S. Roy and S. S. Islam, *Int. J. Biol. Macromol.*, 2015, **79**, 413-422.https://doi.org/10.1016/j.ijbiomac.2015.04.077

35. A. Gucchait, A. Ghosh and A. K. Misra, *Beilstein J. Org. Chem.*, 2019, **15**, 431-436.https://doi.org/10.3762/bjoc.15.37

Appendix. Topology files of new lipids

Topology of PMPE

```
[moleculetype]
; molname      nrexcl
  PMPE         1

[atoms]
; id    type    resnr   residu  atom    cgnr    charge
   1    Qd      1       PMPE    NH3     1       1.0
   2    Qa      1       PMPE    PO4     2       -1.0
   3    Na      1       PMPE    GL1     3       0
   4    Na      1       PMPE    GL2     4       0
   5    C1      1       PMPE    C1A     5       0
   6    C1      1       PMPE    C2A     6       0
   7    C1      1       PMPE    C3A     7       0
   8    C1      1       PMPE    C4A     8       0
   9    C1      1       PMPE    C1B     9       0
  10    C1      1       PMPE    C2B     10      0
  11    C1      1       PMPE    C3B     11      0
  12    C1      1       PMPE    C4B     12      0

[bonds]
;  i  j   funct   length  force.c.
   1  2   1       0.47    1250
   2  3   1       0.47    1250
   3  4   1       0.37    1250
   3  5   1       0.47    1250
   5  6   1       0.465   3000
```

```
  6  7   1         0.42     5000
  7  8   1         0.49     2500
  4  9   1         0.47     1250
  9 10  1          0.47     1250
 10 11             1        0.47     1250
 11 12             1        0.47     1250

[angles]
;  i  j  k         funct    angle    force.c.
  2  3  4          2        120.0    25.0
  2  3  5          2        180.0    25.0
  3  5  6          2        180.0    25.0
  5  6  7          2        180.0    30.0
  6  7  8          2        120.0    20.0
  4  9 10          2        180.0    25.0
  9 10 11          2        180.0    25.0
 10 11 12          2        180.0    25.0


#ifdef POSRES
   [ position_restraints ]
   ; Z postion restraint on lipid head group
     2    1.0     0.0     0.0    POSRES_LHEAD
#endif
```

Topology of PMPG

```
[moleculetype]
; molname    nrexcl
  PMPG       1

[atoms]
; id   type   resnr   residu  atom   cgnr   charge
  1    P4     1       PMPG GL0       1      0.0
  2    Qa     1       PMPG PO4       2      -1.0
  3    Na     1       PMPG GL1       3      0
  4    Na     1       PMPG GL2       4      0
  5    C1     1       PMPG C1A       5      0
  6    C1     1       PMPG C2A       6      0
  7    C1     1       PMPG C3A       7      0
  8    C1     1       PMPG C4A       8      0
  9    C1     1       PMPG C1B       9      0
  10   C1     1       PMPG C2B       10     0
  11   C1     1       PMPG C3B       11     0
  12   C1     1       PMPG C4B       12     0

[bonds]
;  i  j   funct   length   force.c.
   1  2   1       0.47     1250
   2  3   1       0.47     1250
   3  4   1       0.37     1250
   3  5   1       0.47     1250
   5  6   1       0.465    3000
   6  7   1       0.42     5000
   7  8   1       0.49     2500
```

```
  4  9   1        0.47    1250
  9 10  1         0.47    1250
 10 11            1       0.47    1250
 11 12            1       0.47    1250

[angles]
;  i  j  k        funct   angle   force.c.
  2  3  4         2       120.0   25.0
  2  3  5         2       180.0   25.0
  3  5  6         2       180.0   25.0
  5  6  7         2       180.0   30.0
  6  7  8         2       120.0   20.0
  4  9 10         2       180.0   25.0
  9 10 11         2       180.0   25.0
 10 11 12         2       180.0   25.0


#ifdef POSRES
   [ position_restraints ]
   ; Z postion restraint on lipid head group
     2     1.0     0.0     0.0     POSRES_LHEAD
#endif
```
